# Supplementary material for: Qualitative study to explore UK medical students’ and junior doctors’ experiences of occupational stress and mental health during the COVID-19 pandemic
Source: BMJ Open. Author manuscript; Available in PMC 2022 Dec 20. (PMC9748513; doi:10.1136/bmjopen-2022-065639)
Supplement: Online Supplemental Appendix 1 [file EMS158475-supplement-Online_Supplemental_Appendix_1.pdf]

Interview Guide

A qualitative study to explore UK medical students' and junior doctors' experiences of occupational stress and mental health during the COVID-19 pandemic.

Warm up questions

How are you today?

How has work/university been recently?

Have you got any plans this summer?

Main Questions

Experiences of occupational stress

Can you describe a time when you have experienced work-related stress?

Do you make any conscious efforts to reduce work-related stress?

Have you ever felt as though work-related stress has had an impact on your mental wellbeing?

Do you feel as though you are under more pressure than other occupations/degree subjects?

How has the pandemic influenced your experiences of stress/ work stress?

Has the pandemic limited your ability to manage your stress?

Is there anything that you think has been helping your mental health and wellbeing during the pandemic?

Is there anything you have done to try to relieve stress during the pandemic?

Attitudes towards mental illness

Do you feel as though working in the health profession increases or reduces your risk of suffering from a mental illness? – Discuss risk factors and protective factors

Would you agree with the perception that only certain people suffer from mental illnesses? If so, what type of person? Any particular personality traits/ behaviours

How do you feel colleagues would view a co-worker taking leave for mental ill health compared to a physical condition? Why may this be the case?

Barriers to disclosure

Hypothetically if you were suffering from mentally ill health, would there be any factors that may prevent you from disclosing it?

Would you be concerned at all about job stability if you were disclosing a mental illness to a manager or colleagues?

Would you be concerned about experiencing stigma from co-workers if you disclosed a mental illness?

If you were suffering from a mental illness what support networks would be available for you? What would be your preferred route?

Would access to help services be limited by the COVID pandemic?

Cool down questions

Did you find everything okay with the interview?

Do you have any questions or anything you'd like to clarify?

Are you doing anything after this?

Thank participants for their time and for meeting with me.
