# Supplementary material for: Qualitative study to explore UK medical students’ and junior doctors’ experiences of occupational stress and mental health during the COVID-19 pandemic
Source: BMJ Open. 2022 Dec 12;12(12):e065639. doi: 10.1136/bmjopen-2022-065639 (PMC9748513; doi:10.1136/bmjopen-2022-065639)
Supplement: Supplementary file 1 [file bmjopen-2022-065639.draft_revisions.pdf]

BMJ Open is committed to open peer review. As part of this commitment we make the peer review history of every article we publish publicly available.

When an article is published we post the peer reviewers' comments and the authors' responses online. We also post the versions of the paper that were used during peer review. These are the versions that the peer review comments apply to.

The versions of the paper that follow are the versions that were submitted during the peer review process. They are not the versions of record or the final published versions. They should not be cited or distributed as the published version of this manuscript.

BMJ Open is an open access journal and the full, final, typeset and author-corrected version of record of the manuscript is available on our site with no access controls, subscription charges or pay-per-view fees (http://bmjopen.bmj.com).

If you have any questions on BMJ Open's open peer review process please email info.bmjopen@bmj.com

BMJ Open

Medical students and junior doctors' perceptions and experiences of occupational stress and mental health during the COVID-19 pandemic

Journal:	*BMJ Open*
Manuscript ID	bmjopen-2022-065639
Article Type:	Original research
Date Submitted by the Author:	14-Jun-2022
Complete List of Authors:	Tawse, James; University of Glasgow College of Science and Engineering; Alder Hey Children's Hospital<br>Demou, Evangelia; University of Glasgow College of Medical Veterinary and Life Sciences, MRC/CSO Social and Public Health Sciences Unit
Keywords:	MENTAL HEALTH, COVID-19, MEDICAL EDUCATION & TRAINING, OCCUPATIONAL & INDUSTRIAL MEDICINE, Health & safety < HEALTH SERVICES ADMINISTRATION & MANAGEMENT

SCHOLARONE™
Manuscripts

I, the Submitting Author has the right to grant and does grant on behalf of all authors of the Work (as defined in the below author licence), an exclusive licence and/or a non-exclusive licence for contributions from authors who are: i) UK Crown employees; ii) where BMJ has agreed a CC-BY licence shall apply, and/or iii) in accordance with the terms applicable for US Federal Government officers or employees acting as part of their official duties; on a worldwide, perpetual, irrevocable, royalty-free basis to BMJ Publishing Group Ltd ("BMJ") its licensees and where the relevant Journal is co-owned by BMJ to the co-owners of the Journal, to publish the Work in this journal and any other BMJ products and to exploit all rights, as set out in our licence.

The Submitting Author accepts and understands that any supply made under these terms is made by BMJ to the Submitting Author unless you are acting as an employee on behalf of your employer or a postgraduate student of an affiliated institution which is paying any applicable article publishing charge ("APC") for Open Access articles. Where the Submitting Author wishes to make the Work available on an Open Access basis (and intends to pay the relevant APC), the terms of reuse of such Open Access shall be governed by a Creative Commons licence – details of these licences and which Creative Commons licence will apply to this Work are set out in our licence referred to above.

Other than as permitted in any relevant BMJ Author's Self Archiving Policies, I confirm this Work has not been accepted for publication elsewhere, is not being considered for publication elsewhere and does not duplicate material already published. I confirm all authors consent to publication of this Work and authorise the granting of this licence.

For peer review only

Medical students and junior doctors' perceptions and experiences of occupational stress and mental health during the COVID-19 pandemic

James Tawse[1,2*], Evangelia Demou[3]

1. College of Social Sciences, University of Glasgow, Glasgow, UK

2. Alder Hey Children's Hospital, Liverpool, UK

3. MRC/CSO Social and Public Health Sciences Unit, Institute of Health and Wellbeing, University of Glasgow, Glasgow, UK

Corresponding author:
Mr James Tawse
Alder Hey Children's Hospital,
Liverpool,
UK
Email: james.tawse@alderhey.nhs.uk

For peer review only

Word count: 3,906

Abstract

Objectives: This study aimed to explore the occupational experiences of medical students and junior doctors working during the COVID-19 pandemic. In particular, the research sought to identify factors which mediated work-stress; barriers to disclosing mental health problems; and levels of support medical students and junior doctors received during the pandemic.

Design: This study was a form of thematic analysis and adopted an inductive, 'bottom-up' approach, in which coded categories were derived from rich, descriptive data.

Setting: Semi-structured interviews were conducted with UK based medical students and junior doctors. Interviews were recorded and analysis was done by coding salient quotes into themes.

Participants: The final sample consisted of 7 junior doctors and 8 medical students. The inclusion criteria for the study were junior doctors working in the UK, defined as doctors who graduated from medical school in the last five years, and who were currently enrolled on a medical training programme. Medical students were only included if they were studying at universities in the UK and were in their later years of study, from 4th year onwards.

Results: High levels of occupational stress were identified which were exacerbated by COVID-19. In particular, a number of organisational difficulties associated with the pandemic compounded participants' experiences of work-stress. Participants recognised progress towards promoting and managing mental health within the profession but may still be reluctant to access support services. Barriers to disclosure included fear of stigmatisation, concerns about adding to colleagues' workloads, lack of clarity about career implications and mistrust of occupational health services.

For peer review only

Conclusions: Whilst attitudes towards mental health have improved, medical students and junior doctors may still avoid seeking help. Given the immense pressures faced during the COVID-19 pandemic, it is imperative that extra measures are implemented to minimise work-stress, encourage help-seeking behaviours and promote supportive work cultures.

Article Summary

Strengths and limitations of this study:

- This research captured medical students and junior doctors' experiences of working during the summer of 2021; immediately after the first year of the pandemic and alongside rising rates of COVID in the UK, ongoing restrictions to social contact and unprecedented pressure on health services.
- This study accessed a unique cohort of medical students and junior doctors, who did not receive standard training opportunities and provides an understanding of how these groups coped, how well they were supported and what more could be done to promote their wellbeing in the event of future stressful events for health and educational services.
- The FY1s in the study, who were the majority of the junior doctors sampled (5/7), entered the profession during COVID-19 and consequently, it was difficult for this cohort to contextualise their experiences of occupational stress during the pandemic.
- The representativeness and transferability of the findings may be limited, as most participants were males and of a white-British ethnicity.

Keywords: medical student, junior doctor, mental health, occupational stress, COVID-19

For peer review only

Introduction

Education and health systems worldwide have had to radically readjust their working practices to continue functioning during the COVID-19 pandemic. For medical students, this has meant adapting to online learning methods, the discontinuation of clinical placements, novel examination formats and the cancellation of medical electives [1]. For many medical students, studying during the pandemic has been associated with decreased engagement, poorer academic performance and increased feelings of isolation, boredom and anxiety [2]. A perceived lack of support from peers and institutions during this time has led to decreased motivation, lower assessments of quality of life, and heightened stress among medical students [2]. On top of this, the discontinuation of clinical-based attachments could have negative implications for medical students' interpersonal and practical skills, confidence, and ability to work as future doctors [1 3]. Medical students' mental health has suffered during the COVID-19 pandemic, as evidenced by an increasing prevalence of stress and fatigue [4], as well as a 61% rise in rates of general anxiety disorder and a 70% rise in symptoms of major depressive disorder when compared to pre-pandemic figures [5].

Junior doctors have also had to manage a number of additional pressures during the pandemic, including heavy workloads, large death tolls and the fear of contracting the virus [6]. Moreover, junior doctors' resilience has been hindered by loss of social support, infection of friends and family, as well as drastic changes to working practices [7]. On top of this, in the United Kingdom, governing bodies allowed final year medical students to graduate 6-8 weeks early from medical school to join the frontline in fighting against the pandemic [8]. This raises questions about this specific group of newly graduated doctors' preparedness and confidence to enter into the profession, under very difficult circumstances, and is concerning

For peer review only

given that less prepared trainees make more medical errors and suffer from higher rates of burnout [9].

Whilst medical students and trainee doctors have faced significant work-related stress and mental health challenges throughout the pandemic, their access to care may have been obstructed by a number of factors. Access to mental health services has been limited during the pandemic due to staff shortages, a significant rise in emergency cases and the creation of long patient waiting lists [10 11]. Moreover, stigmatisation of mental health problems, concerns about career implications, fear of adding to colleagues' workloads and inadequate support systems have been identified as barriers to help-seeking amongst these groups in previous works [12 13].

The mental health burden of medical students and junior doctors working during the pandemic has been the subject of interest in quantitative research studies [4 6]; but there has been a paucity of in-depth qualitative research in this area. This study aims to develop a greater understanding of the work-stressors experienced by medical students and junior doctors during the pandemic, explore their barriers to accessing care for mental health problems, and assess how educational and health services have adapted their systems to support medical students and junior doctors during the COVID-19 pandemic.

Methods

Participant recruitment

The inclusion criteria for the study were junior doctors working in the UK, defined as doctors who graduated from medical school in the last five years, and who were currently enrolled on a medical training programme. Medical students were only included if they were studying at

For peer review only

universities in the UK and were in their later years of study, from 4th year onwards. An initial sample of participants were recruited through personal contacts and acquaintances of the lead researcher. Following this, participants were recruited using snowball sampling.

Once sampled, participants were contacted to arrange a date and time to conduct the interview. Prior to arranging interviews, participants were made aware of the requirements and purposes of the research, as well as several ethical assurances, so that they could make an informed decision on participation. At the start of the interview, participants were reminded of their ethical rights and briefed on the study. Following this, participants responded to demographic questions, before proceeding to the interview questions.

Semi-structured, one-on-one interviews were conducted with participants over Zoom. For this study, a semi-structured approach meant that participants could express themselves freely, whilst keeping the focus of conversation directed towards the research questions [14]. One-on-one interviews were selected as they enable a greater sense of trust and rapport to be established between participant and researcher [15], which is vital when exploring sensitive topics. Finally, utilising an online video-chat platform provided an optimal alternative to face-to-face interviews, allowing high-quality interviews to be carried out with participants working in a range of locations throughout the UK [16]. Recordings of interviews were taken using the built-in tool on the Zoom application, for later verbatim transcription. After transcription the recordings were erased. The interviews lasted an average of 36 minutes, ranging from 25 to 52 minutes.

Ethical approval was obtained from the University of Glasgow, School of Social & Political Sciences committee (PGT/SPS/2021/035/GLOB).

For peer review only

Patient and public involvement

Patients or the public were not involved in the design and conduct of this study.

Data analysis methodology

An inductively driven thematic analysis of the interview data was undertaken, in accordance with Braun and Clarke's six stage model [17]. The dependability of the findings was assured by conducting regular meetings with the second researcher (ED) to produce a coding framework that accurately captured the themes within the data. Analysis took place in parallel with interviews, which meant that the researcher (JT) could closely follow emerging themes and confirm when data saturation had been achieved, thereby providing a more efficient way to sample [18]. The software *NVivo 12* was used to analyse the data.

Results

Participant demographic information and the thematic map of the findings are presented in Table 1 and Figure 1. The final sample consisted of 15 participants (8 medical students, 7 junior doctors). Out of the 8 medical students, 3 were in their 4th year of study, whilst 5 were in their 5th year of study. With regards to the junior doctors, 5 were in their first year of postgraduate work (FY1), whilst 2 were in their 3rd year (FY3). The participants were working across the UK (Table 1). 80% of the sample were male and the majority were of a White-British ethnic background (86.7%).

For peer review only

Table 1: Sample characteristics

	Frequency	Percent
Training level		
4th year student	3	20%
5th year student	5	33.3%
Junior doctor, foundation year 1 (FY1)	5	33.3%
Junior doctor, foundation year 3 (FY3)	2	13.3%
Total	15	100.0%
Place of work		
Scotland	4	26.7%
North-West England	6	40%
North-East England	2	13.3%
Midlands England	2	13.3%
South-West England	1	6.7%
Total	15	100%
Gender identity		
Male	12	80%
Female	3	20%
Total	15	100.0%
Ethnicity		
White-British	13	86.7%
Asian-British	2	13.3%
Total	15	100.0%

For peer review only

[Insert Figure 1]

Occupational stress inherent to medicine

Sizeable workloads in medicine were a significant stressor amongst participants and many reported an increased prevalence of symptoms of depression and anxiety in the build-up to exams. Some participants felt that their work commitments were more excessive than other university degree subjects or occupational roles, and that this might increase their psychiatric burden.

> *"I lived with non-medics this year and I was quite surprised by how little they did (...), I think when you have to turn up to placement 9-5 every day... I think that pressure drains you and you do get worn down by it" (#3, 5th year student).*

All participants stated that practising medicine can be emotionally challenging, as reflected below from a final year medical student. Moreover, participants remarked that personality traits common to medics, such as competitiveness, perfectionism, self-criticism and a tendency toward workaholism, can be maladaptive in terms of their wellbeing. Despite this, all participants stated that resilience to stress was particularly high amongst medics, and that this protected their occupational group against mental health difficulties.

> *"When you're looking at the clinical side, I do think it is particularly challenging, for example when you're put in situations where you're watching someone die or trying to save someone's life" (#2, 5th year student).*

For peer review only - http://bmjopen.bmj.com/site/about/guidelines.xhtml

For peer review only

"I do think we are quite a resilient group of people, because you kind of have to be, and it's something that just happens to you when you're in medical school and you start working" (#4, FY1).

Organisational challenges associated with the COVID-19 pandemic

A majority of medical students suggested that changes to academic structure had compounded aspects of their education as well as their mental burden during the pandemic. Participants explained how online learning mechanisms provided fewer opportunities for social interaction, led to feelings of frustration and decreased their motivation to study. Simultaneously, changes to course structure placed some participants under heightened time pressures, exacerbating their experiences of occupational stress.

"I much prefer face-to-face because there's much more interaction and it's so much easier to engage, and so much more sociable. You kind of lose motivation when you're just staring at a screen all day in a room by yourself" (#2, 5th year student).

Concerns around preparedness were described, specifically where aspects of education had been impaired or missed altogether. This point is particularly salient for the sub-cohort of junior doctors who graduated early to offer their services during the pandemic. Despite this, a number of junior doctors suggested that even without the pandemic, they would have felt underprepared for the step up from medical school to a qualified doctor.

"I've still never worked in the Emergency Department (ED), I've never even been there as a medical student, so when it eventually comes to me being in ED, I'll definitely feel less prepared in that regard" (#5, FY1).

For peer review only

> *"You get to the point in medical school where you've done your exams and you're going to be as ready as you will be" (#4, FY1).*

Another source of stress, which particularly pertained to the junior doctors, was resourcing problems within healthcare. For example, staffing problems left junior doctors feeling overburdened which compounded their experiences of work stress and impacted upon the quality of care they could provide. In some cases, a shortage of personal protective equipment (PPE) led to concerns around risk of infection and personal health and safety. Moreover, participants suggested that a lack of resources including ventilators and hospital beds had created intensely stressful situations, where staff would regularly be making life and death decisions about who should receive care.

> *"The consultant came back and kind of said... you know, 'Have you done this? Have you done this? Has this happened?' and I was just oblivious to it. It was a particularly understaffed ward in a time when the hospital was understaffed" (#4, FY1).*

> *"That's quite hard knowing that you're sort of holding off optimal treatment for a patient because you don't have beds. Yeah, that was horrible actually, because then equally you've then got families, saying 'well, why aren't we escalating?'" (#6, FY1).*

Medical cultures surrounding work-stress and mental health

Participants discussed cultural norms regarding occupational stress and mental health within the health service. In some cases, participants suggested that work cultures within medicine can block access to mental health services and can perpetuate presenteeism behaviours. For

For peer review only - http://bmjopen.bmj.com/site/about/guidelines.xhtml

example, some participants felt that it may be particularly difficult to disclose a mental health problem whilst practising medicine, due to stigmatisation. More specifically, concerns were raised that peers, tutors or supervisors may perceive them as being mentally weak, or lacking the resilient personality required to practice medicine, if they were to take time off for mental health difficulties.

> *"Anyone that works does this, but particularly people that work in the NHS, you feel you have a duty to go to work, you know even if you're at death's door you go into work" (#4, FY1).*

> *".... I was worried that you know, if they do think that I'm not mentally 100% then I can't be doing this, because I can't handle the stress" (#13, 4th year student).*

All of the participants stated that attitudes towards mental health and disclosure have improved in recent times and this reflected broader perceptions and public awareness of the importance of good mental health. Moreover, participants agreed that recognition of mental health had acutely improved during the pandemic, as evidenced below.

> *"The general consensus is that the kind of historic or traditional view of that is kind of 'pick yourself and get on with it', whereas I think over the last 12 months, but also over the last decade, there's definitely been a shift in acknowledging that health care professionals need to take care of themselves first and foremost" (#1 , 5th year student).*

A number of participants indicated that they would not feel comfortable approaching some senior doctors within their hospitals for mental health support. Despite this, interactions with

For peer review only

senior doctors were generally very positive and participants felt that they were helpful and accessible.

"All through medical school you're taught about these really scary surgeons and consultants that won't take your answers but, on the whole, they've all been really supportive" (#6, FY1).

A recurrent pattern in the data was that junior doctors worried about burdening colleagues with extra work if they were to take time off, mainly due to significant staff shortages during the pandemic. Despite this, medical students and junior doctors adopted an altruistic perspective, whereby they would feel guilty for adding to their colleagues' workloads but noted that they would be happy to pick up the extra work if their colleagues needed time off for their mental wellbeing.

"That's the good thing about being an FY1, you have such good camaraderie, you know you would do that (pick up the extra work when a colleague is off) for your colleagues, there's a real sense of that amongst the workforce" (#4, FY1).

Sources of personal support & coping during the pandemic

The importance of peer support was unanimously felt by all participants. Many explained how they relied on their peers for informal mental health support and to problem solve difficult situations. A majority of the participants also lived with their course-mates or colleagues, which provided them with additional opportunities to open up about their problems.

For peer review only

"We kind of do it without being labelled a support network, so we all come home from placement and will be like, 'I saw this thing today', and we tell the story, and we maybe don't consciously realise but we're saying it to get it off our chest" (#14, 4th year student).

Beneficial work cultures were identified during the interviews including camaraderie, humour and team dynamics, which cultivated a sense of solidarity whilst working under challenging circumstances. Rewarding aspects of working in the medical profession were also highlighted. In particular, participants suggested that practising medicine had provided them with a sense of purpose and identified the psychological benefits associated with treating patients.

"You are doing a job that is inherently helpful and rewarding and you feel good because of it and when something goes well, that buzz stays with you for a long time" (#14, 4th year student).

At the same time, participants discussed how their usual methods for managing stress had been hindered by the COVID-19 pandemic. Many participants went extended periods without seeing their close friends and family, whilst a number of FY1s had found it difficult to integrate into new work settings.

"COVID's restricted the social side of things and meeting new people. So, essentially work is the only place you get to meet new people and it's only been on a professional basis" (#11, FY1).

For peer review only

Awareness of, and attitudes towards formal support mechanisms

Participants felt that occupational health services had made extra efforts to support their wellbeing during COVID-19. Extra email communications, signposting to mental health services, wellbeing posters around hospitals and psychoeducational talks were some methods identified by participants. Despite this, some participants felt that they would have benefitted from receiving further support and discussed potential strategies. These centred around providing students and doctors with more opportunities for rest and recovery from work, fostering peer support within the workplace and making mental health services more accessible.

"During the pandemic there's been a real focus on mental health for all members of staff, be that porters, nurses, doctors, auxiliary staff or healthcare assistants, and there's been a real effort in reaching out to people to take that time away from the job to kind of think about things a bit more" (#4, FY1).

Of the participants that had previously met with occupational services for their mental health, a majority suggested that they had not found it helpful. Participants felt that occupational services were more concerned about what this meant for their academic progression or work availability, rather than focusing on offering them support. Importantly, some participants felt their negative experiences in dealing with occupational services had discouraged them from accessing help for their mental health in the future.

"It was occupational health, and I had a meeting with her, and she was very, very condescending (...) I think that was quite a rare experience, but it's definitely tainted my view of disclosing mental health issues" (#6, FY1).

For peer review only - http://bmjopen.bmj.com/site/about/guidelines.xhtml

Around half of the sample felt that they would be concerned about potential career implications if they disclosed a mental health problem. Some participants felt this may affect their future employability. Moreover, a couple of participants also suggested being reluctant to disclose a mental health problem to their general practitioner (GP), stating concerns about the confidentiality of their medical record.

> *"It's something that you shouldn't be worried about because it shouldn't be too different to any other health condition, but it would worry me, I think it does worry me a little bit, so when I got the job with the NHS, I didn't put that down" (#14, 4th year student).*

Whilst most participants felt supported by their clinical colleagues, tutors and peers, attitudes towards administrative staff were more variable. Some participants felt underappreciated and inadequately supported by management, suggesting that they lacked insight into the pressures involved in practicing medicine.

> *"The senior doctors will try to help you out and give you advice, but not from the actual hospital management, rota co-ordinators, they have no clue, everything's like a number for them, it's almost like the business side of things so they don't know the pressures of actually working for the hospital" (#11, FY1).*

For peer review only

Discussion

Medical students and junior doctors discussed high levels of occupational stress which had impacted on their mental wellbeing during the COVID-19 pandemic. Various stressors inherent to practising medicine, such as significant workloads and emotional challenges, were identified as well as a number of organisational difficulties associated with the pandemic. Issues pertaining to resourcing, staffing, preparedness and adapting to new training formats had exacerbated participants' work-stress during the pandemic. Medical cultures which promote presenteeism and personality traits including perfectionism, self-criticism, competitiveness and resilience were viewed as influencing factors on participants' work stress. Extra efforts to support the wellbeing of medical students and junior doctors during the pandemic were noted, however, participants also perceived various barriers to accessing support. These included a lack of clarity on the career implications, concerns about adding to colleagues' workloads, stigma within the profession and previous negative experiences engaging with occupational health services.

Participants discussed factors that might prevent them from accessing help for their mental wellbeing, which echoed previous findings where medical cultures of secrecy and distrust were identified, as well as concerns around being perceived as weak [13 19]. Furthermore, participants worried about the career implications of disclosing a mental health problem to occupational and health services, indicating the need for improved clarity and transparency in this process. A previous study on depression, stigma and suicidal ideation in medical students revealed that 53% of respondents who reported depressive symptoms were worried that revealing their illness would be risky for their career [20]. Worryingly, many participants described their negative experiences interacting with occupational services, which deterred them from accessing help for their mental health in the future. Despite this, all of the research

For peer review only

participants recognised significant progress towards promoting and managing mental health within the medical profession. Moreover, participants described how attitudes towards mental health had improved acutely over the last 18 months, which may reflect extra efforts to support medical workers during the COVID-19 pandemic [21]. In line with previous findings [22 23], the medical students and junior doctors noted the importance of peers for support. However, a majority of participants indicated that the pandemic had limited their access to support networks and coping strategies which would usually be beneficial for their wellbeing. Furthermore, participants often felt overburdened by factors relating to organisational structure and perceived a lack of organisational support. This follows from previous works [24 25], in which junior doctors voiced their frustrations on organisational issues such as rotas suddenly changing, having to relocate regularly and feeling undervalued by admin staff. Despite this, participants suggested that working in the medical profession had been somewhat protective for their mental wellbeing during COVID-19, in that it is an inherently helpful, rewarding and sociable profession.

The subject of mental health remains a sensitive one. A strength of this research is that an independent researcher carried out the interviews and analysis, adding an extra layer of privacy protection and strong reassurance of participant confidentiality. Overall, participants were open and were keen to provide their perspectives on the mental health issues affecting their workforce. A further study strength is that it accessed this very unique cohort of medical students and junior doctors working in unprecedented times for the medical profession, therefore adding to the current literature, which is sparse [26]. Despite this, the representativeness and transferability of the findings may be limited, as most participants were males and of a white-British ethnicity. Additionally, whilst snowball sampling proved a useful recruitment technique, selection and response biases cannot be excluded [27]. Another

For peer review only

potential limitation is that the FY1s, who were the majority of the junior doctors sampled (5/7), entered the profession during COVID-19. Consequently, it was difficult for this cohort to contextualise their experiences of occupational stress during the pandemic.

Protective measures should be implemented to minimise occupational stressors for medical students' and junior doctors. This might include ensuring that workers do not stay beyond their scheduled hours, and consistently monitoring and checking on their physical and mental wellbeing [28]. Psychoeducational workshops have also proven useful in enhancing workers' coping skills, competence and resilience [29]. Finally, individuals who need to take time off should feel able to do so and be made aware of the systems in place to support them [21].

Steps must be taken to ensure that comprehensive and accessible occupational health care is available for all medical students and junior doctors. Medical cultures which stigmatise mental health problems and promote presenteeism must be replaced with cultures which promote openness and help-seeking. Furthermore, the career implications of disclosing a mental health problem must be clarified. These issues could be addressed using educational and training interventions such as through the distribution of online articles and videos, handbooks, communication training and self-awareness training [28]. Finally, efforts must be made to promote positive working environments where workers feel valued and supported. The importance of peer support was clear in the research data. Consequently, interventions which foster peer support must be considered. Elsewhere, good practices have included 'buddying-up' schemes to help reduce isolation as well as virtual peer support groups where doctors can openly discuss the emotional impact of their work [21 30].

For peer review only

Given the exploratory nature of this research, larger qualitative and quantitative studies are required to determine the generalisability and transferability of the findings. In particular, studies which investigate perceived differences in mental health problems according to sociodemographic, education and geographical factors are warranted to understand the diverse needs of this workforce. Future studies may benefit from exploring the views of educational, occupational and healthcare management staff, to add other perspectives on the factors affecting this cohort's wellbeing. Further validation of the findings could also be added using observational research in order to provide more impetus for quality improvement interventions to be implemented.

Acknowledgements: The authors thank all the participants for sharing their experiences.

Author contributions: JT - conceived and designed the study; conducted the participant interviews, data analyses and drafted the manuscript; ED –contributed to the interpretation of the results and data analysis and with critical revisions of the manuscript.

Data statement: The data is not currently available for further use.

Ethical approval: Ethical approval was obtained from the University of Glasgow, School of Social & Political Sciences committee (PGT/SPS/2021/035/GLOB). Patients or the public were not involved in the design and conduct of this study.

Funding statement: ED acknowledges funding from the Medical Research Council (MC_UU_00022/2) and the Chief Scientist Office (SPHSU17).

Competing interests: An initial sample of participants were recruited through personal contacts of the lead researcher and the remainder were recruited using snowball sampling. ED declares no competing interests.

For peer review only

References

1. Rainbow S, Dorji T. Impact of COVID-19 on medical students in the United Kingdom. *GERMS* 2020;10(3):240-43. doi: 10.18683/germs.2020.1210

2. Longhurst GJ, Stone DM, Dulohery K, et al. Strength, Weakness, Opportunity, Threat (SWOT) Analysis of the Adaptations to Anatomical Education in the United Kingdom and Republic of Ireland in Response to the Covid-19 Pandemic. *Anat Sci Educ* 2020;13(3):301-11. doi: 10.1002/ase.1967 [published Online First: 2020/04/20]

3. Wilcha RJ. Effectiveness of Virtual Medical Teaching During the COVID-19 Crisis: Systematic Review. *JMIR Med Educ* 2020;6(2):e20963. doi: 10.2196/20963 [published Online First: 2020/10/28]

4. Zis P, Artemiadis A, Bargiotas P, et al. Medical Studies during the COVID-19 Pandemic: The Impact of Digital Learning on Medical Students' Burnout and Mental Health. *Int J Environ Res Public Health* 2021;18(1) doi: 10.3390/ijerph18010349 [published Online First: 2021/01/21]

5. Halperin SJ, Henderson MN, Prenner S, et al. Prevalence of Anxiety and Depression Among Medical Students During the Covid-19 Pandemic: A Cross-Sectional Study. *Journal of Medical Education and Curricular Development* 2021;8:1-7. doi: 10.1177/2382120521991150

6. Pandey U, Corbett G, Mohan S, et al. Anxiety, Depression and Behavioural Changes in Junior Doctors and Medical Students Associated with the Coronavirus Pandemic: A Cross-Sectional Survey. *J Obstet Gynaecol India* 2020:1-5. doi: 10.1007/s13224-020-01366-w [published Online First: 2020/09/30]

7. Pappa S, Ntella V, Giannakas T, et al. Prevalence of depression, anxiety, and insomnia among healthcare workers during the COVID-19 pandemic: A systematic review and meta-analysis. *Brain Behav Immun* 2020;88:901-07. doi: 10.1016/j.bbi.2020.05.026 [published Online First: 2020/05/22]

For peer review only

8. Harvey A. Covid-19: medical schools given powers to graduate final year students early to help NHS. *BMJ* 2020;368:m1227. doi: 10.1136/bmj.m1227 [published Online First: 2020/03/29]

9. Monrouxe LV, Bullock A, Gormley G, et al. New graduate doctors' preparedness for practice: a multistakeholder, multicentre narrative study. *BMJ Open* 2018;8(8):e023146. doi: 10.1136/bmjopen-2018-023146 [published Online First: 2018/08/31]

10. Torjesen I. Covid-19: Mental health services must be boosted to deal with "tsunami" of cases after lockdown. *BMJ* 2020;369:m1994. doi: 10.1136/bmj.m1994 [published Online First: 2020/05/18]

11. Byrne A, Barber R, Lim C. Impact of the COVID-19 pandemic – a mental health service perspective. *Prog Neurol Psychiatry* 2021;25:27-33b.

12. Moll SE. The web of silence: a qualitative case study of early intervention and support for healthcare workers with mental ill-health. *BMC Public Health* 2014;14:138. doi: 10.1186/1471-2458-14-138 [published Online First: 2014/02/11]

13. Waugh W, Lethem C, Sherring S, et al. Exploring experiences of and attitudes towards mental illness and disclosure amongst health care professionals: a qualitative study. *J Ment Health* 2017;26(5):457-63. doi: 10.1080/09638237.2017.1322184 [published Online First: 2017/05/11]

14. Jamshed S. Qualitative research method-interviewing and observation. *J Basic Clin Pharm* 2014;5(4):87-8. doi: 10.4103/0976-0105.141942 [published Online First: 2014/10/16]

15. Dempsey L, Dowling M, Larkin P, et al. Sensitive Interviewing in Qualitative Research. *Res Nurs Health* 2016;39(6):480-90. doi: 10.1002/nur.21743 [published Online First: 2016/07/20]

For peer review only

16. Lobe B, Morgan D, Hoffman KA. Qualitative Data Collection in an Era of Social Distancing. *International Journal of Qualitative Methods* 2020;19 doi: 10.1177/1609406920937875

17. Braun V, Clarke V. Using thematic analysis in psychology. *Qualitative Research in Psychology* 2006;3(2):77-101. doi: 10.1191/1478088706qp063oa

18. Palinkas LA. Qualitative and mixed methods in mental health services and implementation research. *J Clin Child Adolesc Psychol* 2014;43(6):851-61. doi: 10.1080/15374416.2014.910791 [published Online First: 2014/10/29]

19. Bianchi EF, Bhattacharyya MR, Meakin R. Exploring senior doctors' beliefs and attitudes regarding mental illness within the medical profession: a qualitative study. *BMJ Open* 2016;6(9):e012598. doi: 10.1136/bmjopen-2016-012598 [published Online First: 2016/09/18]

20. Schwenk TL, Davis L, Wimsatt LA. Depression, stigma, and suicidal ideation in medical students. *JAMA* 2010;304(11):1181-90. doi: 10.1001/jama.2010.1300 [published Online First: 2010/09/16]

21. BMA. The mental health and wellbeing of the medical workforce – now and beyond COVID-19. 2021

22. Fisher EB, Ayala GX, Ibarra L, et al. Contributions of Peer Support to Health, Health Care, and Prevention: Papers from Peers for Progress. *Ann Fam Med* 2015;13 Suppl 1:S2-8. doi: 10.1370/afm.1852 [published Online First: 2015/08/26]

23. Moir F, Henning M, Hassed C, et al. A Peer-Support and Mindfulness Program to Improve the Mental Health of Medical Students. *Teach Learn Med* 2016;28(3):293-302. doi: 10.1080/10401334.2016.1153475 [published Online First: 2016/04/20]

For peer review only

24. Wainwright E, Fox F, Breffni T, et al. Coming back from the edge: a qualitative study of a professional support unit for junior doctors. *BMC Med Educ* 2017;17(1):142. doi: 10.1186/s12909-017-0978-0 [published Online First: 2017/08/25]

25. Smith SE, Tallentire VR, Pope LM, et al. Foundation Year 2 doctors' reasons for leaving UK medicine: an in-depth analysis of decision-making using semistructured interviews. *BMJ Open* 2018;8(3):e019456. doi: 10.1136/bmjopen-2017-019456 [published Online First: 2018/03/04]

26. Kerins J, Hamilton AL, Pringle J, et al. Exploring the impact of the COVID-19 pandemic on doctors' core workplace needs: a qualitative study of internal medicine trainees in Scotland. *BMJ Open* 2021;11(6):e053506. doi: 10.1136/bmjopen-2021-053506 [published Online First: 2021/07/02]

27. Pannucci CJ, Wilkins EG. Identifying and avoiding bias in research. *Plast Reconstr Surg* 2010;126(2):619-25. doi: 10.1097/PRS.0b013e3181de24bc [published Online First: 2010/08/04]

28. Vizheh M, Qorbani M, Arzaghi SM, et al. The mental health of healthcare workers in the COVID-19 pandemic: A systematic review. *J Diabetes Metab Disord* 2020:1-12. doi: 10.1007/s40200-020-00643-9 [published Online First: 2020/11/03]

29. Johnson J, Simms-Ellis R, Janes G, et al. Can we prepare healthcare professionals and students for involvement in stressful healthcare events? A mixed-methods evaluation of a resilience training intervention. *BMC Health Serv Res* 2020;20(1):1094. doi: 10.1186/s12913-020-05948-2 [published Online First: 2020/11/29]

30. Behrman S, Baruch N, Stegen G. Peer support for junior doctors: a positive outcome of the COVID-19 pandemic? *Future Healthc J* 2020;7(3):e64-e66. doi: 10.7861/fhj.2020-0069 [published Online First: 2020/10/24]

For peer review only

Figure Legends

Figure 1**.** Thematic Map

For peer review only

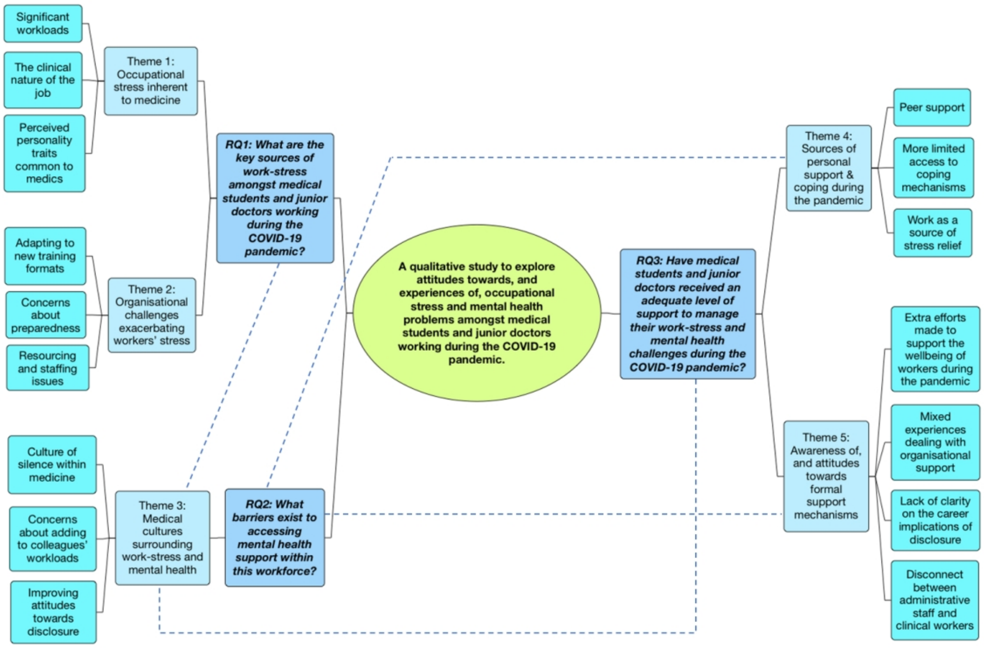

Thematic Map

165x109mm (236 x 236 DPI)

Standards for Reporting Qualitative Research (SRQR)*

http://www.equator-network.org/reporting-guidelines/srqr/

Title and abstract

	Page/line no(s).
**Title** - Concise description of the nature and topic of the study Identifying the study as qualitative or indicating the approach (e.g., ethnography, grounded theory) or data collection methods (e.g., interview, focus group) is recommended	Page 2, Line 3
**Abstract** - Summary of key elements of the study using the abstract format of the intended publication; typically includes background, purpose, methods, results, and conclusions	Page 3, Line 3

Introduction

**Problem formulation** - Description and significance of the problem/phenomenon studied; review of relevant theory and empirical work; problem statement	Page 6, Line 5
**Purpose or research question** - Purpose of the study and specific objectives or questions	Page 7, Line 36

Methods

**Qualitative approach and research paradigm** - Qualitative approach (e.g., ethnography, grounded theory, case study, phenomenology, narrative research) and guiding theory if appropriate; identifying the research paradigm (e.g., postpositivist, constructivist/ interpretivist) is also recommended; rationale**	Page 9, Line 19
**Researcher characteristics and reflexivity** - Researchers' characteristics that may influence the research, including personal attributes, qualifications/experience, relationship with participants, assumptions, and/or presuppositions; potential or actual interaction between researchers' characteristics and the research questions, approach, methods, results, and/or transferability	Page 8, Line 15
**Context** - Setting/site and salient contextual factors; rationale**	Page 7, Line 30
**Sampling strategy** - How and why research participants, documents, or events were selected; criteria for deciding when no further sampling was necessary (e.g., sampling saturation); rationale**	Page 8, Line 8
**Ethical issues pertaining to human subjects** - Documentation of approval by an appropriate ethics review board and participant consent, or explanation for lack thereof; other confidentiality and data security issues	Page 9, Line 10
**Data collection methods** - Types of data collected; details of data collection procedures including (as appropriate) start and stop dates of data collection and analysis, iterative process, triangulation of sources/methods, and modification of procedures in response to evolving study findings; rationale**	Page 8, Line 40

For peer review only - http://bmjopen.bmj.com/site/about/guidelines.xhtml

**Data collection instruments and technologies** - Description of instruments (e.g., interview guides, questionnaires) and devices (e.g., audio recorders) used for data collection; if/how the instrument(s) changed over the course of the study	Page 8, Line 40
**Units of study** - Number and relevant characteristics of participants, documents, or events included in the study; level of participation (could be reported in results)	Page 9, Line 43
**Data processing** - Methods for processing data prior to and during analysis, including transcription, data entry, data management and security, verification of data integrity, data coding, and anonymization/de-identification of excerpts	Page 8, Line 56
**Data analysis** - Process by which inferences, themes, etc., were identified and developed, including the researchers involved in data analysis; usually references a specific paradigm or approach; rationale**	Page 9, Line 22
**Techniques to enhance trustworthiness** - Techniques to enhance trustworthiness and credibility of data analysis (e.g., member checking, audit trail, triangulation); rationale**	Page 9, Line 24

Results/findings

**Synthesis and interpretation** - Main findings (e.g., interpretations, inferences, and themes); might include development of a theory or model, or integration with prior research or theory	Page 11, Line 8
**Links to empirical data** - Evidence (e.g., quotes, field notes, text excerpts, photographs) to substantiate analytic findings	Page 11, Line 24

Discussion

**Integration with prior work, implications, transferability, and contribution(s) to the field** - Short summary of main findings; explanation of how findings and conclusions connect to, support, elaborate on, or challenge conclusions of earlier scholarship; discussion of scope of application/generalizability; identification of unique contribution(s) to scholarship in a discipline or field	Page 19, Line 8
**Limitations** - Trustworthiness and limitations of findings	Page 20, Line 54

Other

**Conflicts of interest** - Potential sources of influence or perceived influence on study conduct and conclusions; how these were managed	Page 5, Line 12
**Funding** - Sources of funding and other support; role of funders in data collection, interpretation, and reporting	Page 5, Line 3

*The authors created the SRQR by searching the literature to identify guidelines, reporting standards, and critical appraisal criteria for qualitative research; reviewing the reference lists of retrieved sources; and contacting experts to gain feedback. The SRQR aims to improve the transparency of all aspects of qualitative research by providing clear standards for reporting qualitative research.

For peer review only

For peer review only - http://bmjopen.bmj.com/site/about/guidelines.xhtml

**The rationale should briefly discuss the justification for choosing that theory, approach, method, or technique rather than other options available, the assumptions and limitations implicit in those choices, and how those choices influence study conclusions and transferability. As appropriate, the rationale for several items might be discussed together.

Reference:

O'Brien BC, Harris IB, Beckman TJ, Reed DA, Cook DA. **Standards for reporting qualitative research: a synthesis of recommendations.** *Academic Medicine*, Vol. 89, No. 9 / Sept 2014 DOI: 10.1097/ACM.0000000000000388

For peer review only

BMJ Open

A qualitative study to explore UK medical students' and junior doctors' experiences of occupational stress and mental health during the COVID-19 pandemic

Journal:	*BMJ Open*
Manuscript ID	bmjopen-2022-065639.R1
Article Type:	Original research
Date Submitted by the Author:	26-Oct-2022
Complete List of Authors:	Tawse, James; University of Glasgow College of Science and Engineering; Alder Hey Children's Hospital<br>Demou, Evangelia; University of Glasgow College of Medical Veterinary and Life Sciences, MRC/CSO Social and Public Health Sciences Unit
**Primary Subject Heading**:	Mental health
Secondary Subject Heading:	Occupational and environmental medicine, Public health, Qualitative research, Medical education and training
Keywords:	MENTAL HEALTH, COVID-19, MEDICAL EDUCATION & TRAINING, OCCUPATIONAL & INDUSTRIAL MEDICINE, Health & safety < HEALTH SERVICES ADMINISTRATION & MANAGEMENT

SCHOLARONE™
Manuscripts

I, the Submitting Author has the right to grant and does grant on behalf of all authors of the Work (as defined in the below author licence), an exclusive licence and/or a non-exclusive licence for contributions from authors who are: i) UK Crown employees; ii) where BMJ has agreed a CC-BY licence shall apply, and/or iii) in accordance with the terms applicable for US Federal Government officers or employees acting as part of their official duties; on a worldwide, perpetual, irrevocable, royalty-free basis to BMJ Publishing Group Ltd ("BMJ") its licensees and where the relevant Journal is co-owned by BMJ to the co-owners of the Journal, to publish the Work in this journal and any other BMJ products and to exploit all rights, as set out in our licence.

The Submitting Author accepts and understands that any supply made under these terms is made by BMJ to the Submitting Author unless you are acting as an employee on behalf of your employer or a postgraduate student of an affiliated institution which is paying any applicable article publishing charge ("APC") for Open Access articles. Where the Submitting Author wishes to make the Work available on an Open Access basis (and intends to pay the relevant APC), the terms of reuse of such Open Access shall be governed by a Creative Commons licence – details of these licences and which Creative Commons licence will apply to this Work are set out in our licence referred to above.

Other than as permitted in any relevant BMJ Author's Self Archiving Policies, I confirm this Work has not been accepted for publication elsewhere, is not being considered for publication elsewhere and does not duplicate material already published. I confirm all authors consent to publication of this Work and authorise the granting of this licence.

For peer review only

A qualitative study to explore UK medical students' and junior doctors' experiences of occupational stress and mental health during the COVID-19 pandemic

James Tawse[1,2*], Evangelia Demou[3]

1. College of Social Sciences, University of Glasgow, Glasgow, UK

2. Alder Hey Children's Hospital, Liverpool, UK

3. MRC/CSO Social and Public Health Sciences Unit, Institute of Health and Wellbeing, University of Glasgow, Glasgow, UK

Corresponding author:
Mr James Tawse
Alder Hey Children's Hospital,
Liverpool,
UK
Email: james.tawse@alderhey.nhs.uk

For peer review only

Word count: 3,979

Abstract

Objectives: This qualitative study aimed to explore the occupational experiences of medical students and junior doctors working during the COVID-19 pandemic. In particular, the research sought to identify factors which mediated work stress; barriers to disclosing mental health problems; and levels of support medical students and junior doctors received during the pandemic.

Design: This study was a form of thematic analysis and adopted an inductive, 'bottom-up' approach, in which coded categories were derived from rich, descriptive data.

Setting: Semi-structured interviews were conducted online with UK based medical students and junior doctors. Interviews were recorded, and analysis was done by coding salient quotes into themes.

Participants: The final sample consisted of 7 junior doctors and 8 medical students, during the summer of 2021.

Results: High levels of occupational stress were identified which were exacerbated by COVID-19. A number of organisational difficulties associated with the pandemic compounded participants' experiences of work stress. Participants recognised progress toward promoting and managing mental health within the profession but may still be reluctant to access support services. Barriers to disclosure included fear of stigmatisation, concerns about adding to colleagues' workloads, lack of clarity about career implications and mistrust of occupational health services.

Conclusions: Whilst attitudes towards mental health have improved, medical students and junior doctors may avoid seeking help. Given the immense pressures faced by health services, it is imperative that extra measures are implemented to minimise work-stress, encourage help-seeking behaviours and promote supportive work cultures.

For peer review only - http://bmjopen.bmj.com/site/about/guidelines.xhtml

Article Summary

Strengths and limitations of this study:

- This research captured medical students and junior doctors' experiences of working during the summer of 2021; alongside rising rates of COVID in the UK, ongoing restrictions to social contact and unprecedented pressure on health services.
- This study accessed a unique cohort of medical students and junior doctors, who did not receive standard training opportunities.
- Thematic analysis was conducted on the interview data, facilitating the development of comprehensive and insightful themes.
- The representativeness and transferability of the findings may be limited, as most participants were males and of a white-British ethnicity.

Keywords: medical student, junior doctor, mental health, occupational stress, COVID-19

For peer review only - http://bmjopen.bmj.com/site/about/guidelines.xhtml

Introduction

Coronavirus disease (COVID-19) is one of the most severe international health problems in the last decades [1]. Communities worldwide suffered directly from high infection rates associated with increased mortality, and indirectly through measures to contain the spreading of the virus [2]. In the UK, education, workplaces and health systems had to radically adjust their working practices to adhere to social distancing, safety, and health guidelines[3 4]. For medical students and junior doctors, the pandemic presented an unexpected, disruptive external shock which created uncertainty and distress [5 6].

Since the onset of the pandemic, medical students experienced drastic changes to their academic curricula[7]. Most medical schools shifted to online learning with a reduction or even suspension of practical teaching [5]. Medical students were faced with the discontinuation of clinical placements, novel examination formats and studying in isolation[8]. In response to the public health crisis, medical students across the UK volunteered in hospitals and General Practices, to support shortages in medical staff [7]. However, this placed volunteers at an increased risk of infection, and presented challenges to provide students with adequate supervision [9]. Furthermore, UK governing bodies allowed final year medical students to graduate 6-8 weeks early from medical school to join the frontline in fighting against the pandemic [10]. This raises questions about this group of newly graduated doctors' preparedness to enter the profession, under difficult circumstances, and is concerning given that less prepared trainees make more medical errors and suffer from higher rates of burnout [11].

Junior doctors working to fight COVID-19 reported stress and distress due to the strain on healthcare systems [6]. Such stressors included understaffing, increased workload and uncertainty due to major changes to working practices [12 13]. On top of this, healthcare

For peer review only - http://bmjopen.bmj.com/site/about/guidelines.xhtml

professionals' resilience was hindered by loss of support, infection of friends and family, as well as exposure to unprecedented levels of suffering and illness, both of colleagues and patients[14].

Before the pandemic, concerns had already been raised about medical students and junior doctors' wellbeing [15-18]. Research had consistently shown that medical students and junior doctors experience significant levels of work-stress and burnout, as well as high rates of mental health problems and suicide when compared to the general population [18-21].
In recent times, systemic issues within healthcare have compounded workers' mental health difficulties [22-24]. Poor working conditions, inadequate support mechanisms, and a lack of flexibility have contributed towards record numbers of trainee doctors leaving the profession altogether [25].

The mental health burden of healthcare workers and medical students working during the pandemic has been the subject of interest in quantitative research studies [12 26-28]; but there has been less qualitative research on junior doctors [6 13 29-33] and medical students in particular [34]. As such, this paper can offer valuable insights into these groups' relative stress, mental wellbeing and access to support during this difficult transitionary period from student to doctor [35], and in unprecedented circumstances. This qualitative study captures the voices of these groups at a distinct and testing time, amongst rising rates of COVID-19 in the summer of 2021 and the long-term impact of COVID-19 on healthcare workers' wellbeing remains unclear [36 37]. Consequently, continued efforts to protect medical students' and junior doctors' wellbeing are warranted. In doing so, retention issues may be addressed, which threaten patient safety, and the future of health services [25].

For peer review only - http://bmjopen.bmj.com/site/about/guidelines.xhtml

This study aimed to explore medical students' and junior doctors' experiences of occupational stress and mental health during the COVID-19 pandemic in the UK. Specifically, this study addressed the following three research questions. The research questions informed the study and the interview guide (Appendix 1), they were not directly asked to the participants.

RQ1: What are the key sources of occupational stress amongst medical students and junior doctors working during the COVID-19 pandemic?

RQ2: What barriers exist to accessing mental health support within this workforce?

RQ3: Have medical students and junior doctors received sufficient support to manage their work stress and mental health challenges during the COVID-19 pandemic?

Methods

Participant recruitment

The inclusion criteria for the study were medical students and junior doctors working in the UK, during the summer of 2021. For the present study, 'junior doctor' refers to a doctor who graduated from medical school in the last five years, and who's currently enrolled on a medical training programme. Medical students were only invited to take part if they were in 4th year onwards. This cut-off was selected as these students have experience of clinical placement, and they faced significant disruption to their training, in close proximity to qualification. A combination of sampling strategies were used to recruit participants [38]. Initially, purposeful sampling of participants was implemented, and participants were recruited through personal contacts and acquaintances of the lead researcher. Following this, participants were recruited using snowball sampling [39 40].

For peer review only - http://bmjopen.bmj.com/site/about/guidelines.xhtml

Once sampled, participants were contacted to arrange one-to-one interviews and interviews were arranged for a time that suited the participants [6]. Before starting the interview, participants were made aware of the requirements and purposes of the research, the identity of the researcher, and how the results would be used [41 42]. Participants were reminded of their ethical rights surrounding confidentiality, data protection and their right to withdraw at any point [43]. A safety plan was in place, to ensure that support could be provided in the event of a concerning disclosure [6 44]. Following this, participants made an informed decision on their participation.

Semi-structured, one-on-one interviews were conducted online (Appendix 1). Interviews took place over Zoom™, to adhere to the social distancing guidelines in place (July-August 2021) [45]. Recordings of interviews were taken using the built-in tool on Zoom™, for later verbatim transcription. After transcription, the recordings were erased. Interviews lasted an average of 36 minutes (range: 25 to 52 minutes).

The Standards for Reporting Qualitative Research reporting guidelines were utilised (Appendix 2) [46]. Ethical approval was obtained from the University of Glasgow, School of Social & Political Sciences Committee (PGT/SPS/2021/035/GLOB).

Patient and public involvement

Patients or the public were not involved in the design and conduct of this study.

Data analysis methodology

An inductively driven thematic analysis of the interview data was undertaken, in accordance with Braun and Clarke's six-stage model [47]. The dependability of the findings was assured by

For peer review only - http://bmjopen.bmj.com/site/about/guidelines.xhtml

For peer review only

conducting regular meetings with the second researcher (ED) to produce a coding framework that accurately captured the emerging themes. Data analysis took place in parallel with interviews, which meant that the researcher could closely follow emerging themes and confirm when no new themes were arising [48]. *NVivo 12* was used to analyse the data.

Results

Participant demographic information and the thematic map are presented in Table 1 and Figure 1, respectively. The final sample consisted of 15 participants (8 medical students, 7 junior doctors). Of the medical students, 3 were in their 4th year of study, whilst 5 were in their 5th year of study. With regards to the junior doctors, 5 were in their first year of postgraduate work (FY1), whilst 2 were in their 3rd year (FY3). The participants were working across the UK; 12 were male and the majority (13/15) were of a White-British ethnic background (Table 1).

For peer review only

Table 1: Sample characteristics				
**Participant ID**	**Training level**	**Place of work/study**	**Gender Identity**	**Ethnicity**
**#1**	5th year medical student	North-West England	Male	White-British
**#2**	5th year medical student	North-West England	Male	White-British
**#3**	4th year medical student	North-East England	Male	White-British
**#4**	FY1	Scotland	Male	White-British
**#5**	FY1	Midlands England	Male	White-British
**#6**	FY1	Midlands England	Female	White-British
**#7**	5th year medical student	North-West England	Male	White-British
**#8**	4th year medical student	South-East England	Male	White-British
**#9**	FY1	Scotland	Male	White-British
**#10**	FY3	North-East England	Female	White-British
**#11**	FY1	North-West England	Male	Asian-British
**#12**	5th year medical student	North-West England	Male	White-British
**#13**	5th year medical student	Scotland	Male	White-British
**#14**	4th year medical student	Scotland	Male	White-British
**#15**	FY3	South-East England	Female	Asian-British

For peer review only - http://bmjopen.bmj.com/site/about/guidelines.xhtml

[Insert Figure 1]

Occupational stress inherent to medicine

Sizeable workloads in medicine were a significant stressor amongst participants and many reported an increased prevalence of symptoms of depression and anxiety in the build-up to exams. Some participants felt that their work commitments were more excessive than other degree subjects or occupational roles, and that this might make them more susceptible to poor mental health.

> *"I lived with non-medics this year and I was quite surprised by how little they did (...), I think when you have to turn up to placement 9-5 every day... I think that pressure drains you and you do get worn down by it" (#3, 5th year student).*

All participants stated that practising medicine can be emotionally challenging, as reflected below from a final year medical student.

> *"When you're looking at the clinical side, I do think it is particularly challenging, for example when you're put in situations where you're watching someone die or trying to save someone's life" (#2, 5th year student).*

Moreover, participants remarked that personality traits common to medics, such as competitiveness, perfectionism, self-criticism and a tendency toward workaholism, can be maladaptive in terms of their wellbeing.

For peer review only - http://bmjopen.bmj.com/site/about/guidelines.xhtml

"The people that medicine attracts can be perfectionists where they might be more vulnerable to negative experiences and working in medicine comes with its fair share of those" (#7, 4th year student).

Participants explained that you have to be resilient to practise medicine, to cope with the workload and the emotional aspects of the job. In some cases, participants alluded to a socialisation process which begins upon entry to medical school, where students must claim or develop the resilience that the profession requires.

"I do think we are quite a resilient group of people, because you kind of have to be, and it's something that just happens to you when you're in medical school and you start working" (#4, FY1).

Organisational challenges associated with the COVID-19 pandemic

A majority of medical students suggested that changes to academic structure had compounded aspects of their education as well as their mental burden during the pandemic. Participants explained how online learning mechanisms provided fewer opportunities for social interaction, led to feelings of frustration and decreased their motivation to study.

"I much prefer face-to-face because there's much more interaction and it's so much easier to engage, and so much more sociable. You kind of lose motivation when you're just staring at a screen all day in a room by yourself" (#2, 5th year student).

For peer review only - http://bmjopen.bmj.com/site/about/guidelines.xhtml

Concerns around preparedness were described, specifically where aspects of education had been impaired or missed altogether. This point is particularly salient for the sub-cohort of junior doctors who graduated early to offer their services during the pandemic.

> *"I've still never worked in the Emergency Department (ED), I've never even been there as a medical student, so when it eventually comes to me being in ED, I'll definitely feel less prepared in that regard" (#5, FY1).*

Despite this, several junior doctors suggested that even without the pandemic, they would have felt underprepared for the step up from medical school to a qualified doctor.

> *"You get to the point in medical school where you've done your exams and you're going to be as ready as you will be" (#4, FY1).*

Another source of stress, which particularly pertained to junior doctors, was resourcing problems within healthcare. For example, staffing problems left junior doctors feeling overburdened which compounded their experiences of work stress and impacted upon the quality of care they could provide.

> *"The consultant came back and kind of said... you know, 'Have you done this? Have you done this? Has this happened?' and I was just oblivious to it. It was a particularly understaffed ward in a time when the hospital was understaffed" (#4, FY1).*

In some cases, a shortage of personal protective equipment (PPE) led to concerns around risk of infection and personal health and safety. Moreover, participants suggested that a lack of

For peer review only - http://bmjopen.bmj.com/site/about/guidelines.xhtml

resources including ventilators and hospital beds had created intensely stressful situations, where staff would regularly be making life and death decisions about who should receive care.

> *"That's quite hard knowing that you're sort of holding off optimal treatment for a patient because you don't have beds. Yeah, that was horrible actually, because then equally you've then got families, saying 'well, why aren't we escalating?'" (#6, FY1).*

For peer review only

Medical cultures surrounding work-stress and mental health

Participants discussed cultural norms regarding occupational stress and mental health within the health service. A culture where healthcare workers' schedules and work-life balance were not prioritised was noted. Junior doctors explained that it was common to be asked to stay late or work on your day off at short notice. Participants described experiencing pressure and guilt when being asked to work extra hours and suggested that it was particularly difficult to decline requests during the pandemic.

> *"It's a really difficult one because you just feel like you should, you've got an obligation to do it, and it kind of became the attitude during the pandemic that you weren't doing anything anyway" (#4, FY1).*

In some cases, participants suggested that medical cultures can block access to mental health services and can perpetuate presenteeism behaviours. For example, some participants felt that it may be particularly difficult to disclose a mental health problem whilst practising medicine, due to stigmatisation. Specifically, concerns were raised that peers, tutors or supervisors may

For peer review only - http://bmjopen.bmj.com/site/about/guidelines.xhtml

perceive them as being mentally weak, or lacking the resilient personality required to practice medicine, if they were to take time off for mental health difficulties.

> *"Anyone that works does this, but particularly people that work in the NHS, you feel you have a duty to go to work, you know even if you're at death's door you go into work" (#4, FY1).*

> *"…. I was worried that you know, if they do think that I'm not mentally 100% then I can't be doing this, because I can't handle the stress" (#13, 4th year student).*

All of the participants stated that attitudes towards mental health and disclosure have improved in recent times and this reflected broader perceptions and public awareness of the importance of good mental health. Moreover, participants agreed that recognition of mental health had acutely improved during the pandemic, as evidenced below.

> *"The general consensus is that the kind of historic or traditional view of that is kind of 'pick yourself and get on with it', whereas I think over the last 12 months, but also over the last decade, there's definitely been a shift in acknowledging that health care professionals need to take care of themselves first and foremost" (#1 , 5th year student).*

A number of participants indicated that they would not feel comfortable approaching senior doctors within their hospitals for mental health support. Despite this, interactions with senior doctors were generally positive and participants felt that they were helpful and accessible.

For peer review only

"All through medical school you're taught about these really scary surgeons and consultants that won't take your answers but, on the whole, they've all been really supportive" (#6, FY1).

Junior doctors worried about burdening colleagues with extra work if they were to take time off, mainly due to significant staff shortages during the pandemic. Despite this, medical students and junior doctors adopted an altruistic perspective, whereby they would feel guilty for adding to their colleagues' workloads but noted that they would be happy to pick up the extra work if their colleagues needed time off for their mental wellbeing.

"That's the good thing about being an FY1, you have such good camaraderie, you know you would do that (pick up the extra work when a colleague is off) for your colleagues, there's a real sense of that amongst the workforce" (#4, FY1).

Personal support & coping during the pandemic

The importance of peer support was unanimously felt by all participants. Many relied on their peers for informal mental health support and to problem solve difficult situations. A majority of the participants also lived with their course-mates or colleagues, which provided them with additional opportunities to open up about their problems.

"We kind of do it without being labelled a support network, so we all come home from placement and will be like, 'I saw this thing today', and we tell the story, and we maybe don't consciously realise but we're saying it to get it off our chest" (#14, 4th year student).

For peer review only - http://bmjopen.bmj.com/site/about/guidelines.xhtml

Beneficial work cultures, including camaraderie, humour and team dynamics, cultivated a sense of solidarity whilst working in challenging circumstances. Rewarding aspects of working in the medical profession were also highlighted. In particular, participants suggested that practising medicine had provided them with a sense of purpose and identified the psychological benefits associated with treating patients.

> *"You are doing a job that is inherently helpful and rewarding and you feel good because of it and when something goes well, that buzz stays with you for a long time" (#14, 4th year student).*

Simultaneously, participants discussed how their usual methods for managing stress had been hindered by the COVID-19 pandemic. Many participants described feeling isolated and went extended periods without seeing their close friends and family, whilst several FY1s found it difficult to integrate into new work settings.

> *"When lockdown was happening, I couldn't leave, and I think it got to the point where I was getting quite stressed about if my parents get sick, or my grandparents got sick, I wouldn't be there" (#15, FY3).*

Awareness of formal support mechanisms

Participants felt that occupational health services had made extra efforts to support their wellbeing during COVID-19 (Box 1, Quote 1). Extra email communications, signposting to mental health services, wellbeing posters around hospitals and psychoeducational talks were some identified methods.

For peer review only - http://bmjopen.bmj.com/site/about/guidelines.xhtml

However, some participants felt that they would have benefitted from receiving further support . These centred around providing students and doctors with more opportunities for rest and recovery, fostering peer support within the workplace and making mental health services more accessible (Box 1, Quote 2).

Of the participants that had previously met with occupational services for their mental health, a majority suggested that they had not found it helpful. Participants felt that occupational services were more concerned about what this meant for their academic progression or work availability, rather than on offering them support. Some participants felt their negative experiences in dealing with occupational health services had discouraged them from accessing help in the future (Box 1, Quote 3).

Around half of the sample indicated that they would be concerned about career implications if they disclosed a mental health problem (Box 1, Quote 4). Some participants felt this may affect their future employability. Moreover, a couple of participants indicated reluctance to disclose a mental health problem to their general practitioner, stating concerns about the confidentiality of their medical record.

Whilst most participants felt supported by their clinical colleagues, tutors and peers, attitudes towards administrative staff were more variable. Participants reported feeling underappreciated and inadequately supported by management, suggesting that they lacked insight into the pressures involved in practicing medicine (Box 1, Quote 5).

For peer review only

***Quote 1**: "During the pandemic there's been a real focus on mental health for all members of staff, be that porters, nurses, doctors, auxiliary staff or healthcare assistants, and there's been a real effort in reaching out to people" (#4, FY1).*

***Quote 2:** "I think X university do it, they have mental health days where you can sort of, no questions asked, 'I'm not going in today', and you can just email them and say that, and you have a certain number throughout the year" (#8, 5th year student).*

***Quote 3**: "It was occupational health, and I had a meeting with her, and she was very, very condescending (...), I think that was quite a rare experience, but it's definitely tainted my view of disclosing mental health issues" (#6, FY1).*

***Quote 4:** "It's something that you shouldn't be worried about because it shouldn't be too different to any other health condition, but it would worry me, I think it does worry me a little bit, so when I got the job with the NHS, I didn't put that down" (#14, 4th year student).*

***Quote 5:** "The senior doctors will try to help you out and give you advice, but not from the actual hospital management, rota co-ordinators, they have no clue, everything's like a number for them, it's almost like the business side of things so they don't know the pressures of actually working for the hospital" (#11, FY1).*

Box 1. Participants quotes from Theme 5 : Awareness of formal support mechanisms

For peer review only - http://bmjopen.bmj.com/site/about/guidelines.xhtml

Discussion

Medical students and junior doctors discussed high levels of occupational stress which impacted on their wellbeing during the COVID-19 pandemic. Various stressors inherent to practising medicine, such as significant workloads and emotional challenges, were identified as well as organisational difficulties associated with the pandemic. Issues pertaining to resourcing, staffing, preparedness and adapting to new training formats exacerbated participants' work-stress during the pandemic. Extra efforts to support medical students and junior doctors during the pandemic were noted, however, participants also perceived barriers to accessing mental health support. These included a lack of clarity on their career implications, concerns about adding to colleagues' workloads, stigma in the profession and previous negative experiences with occupational health services.

Medical cultures which promote presenteeism and personality traits including perfectionism, self-criticism, and competitiveness were viewed as influencing participants' work stress. Previously, a 'hidden curriculum' within medical training has been described, where trainees are indoctrinated with social norms that may be maladaptive [22 24]. These norms place medical trainees under pressure to accept insufficient working conditions [24 25 49 50], or feel guilty for letting patients down, and burdening their colleagues with extra work if they themselves need time off [6 51 52]. At the same time, doctors receive validation from their peers for going above and beyond for their patients, producing an environment which sustains burnout [24].

Participants discussed factors that might prevent them from accessing help for their mental wellbeing, which echoed previous findings where medical cultures of secrecy and distrust were identified, as well as concerns around being perceived as weak [53 54]. Furthermore, participants worried about the career implications of disclosing a mental health problem to

For peer review only - http://bmjopen.bmj.com/site/about/guidelines.xhtml

occupational and health services, indicating the need for improved clarity and transparency in this process. A previous study on depression, stigma and suicidal ideation in medical students revealed that 53% of respondents who reported depressive symptoms worried that revealing their illness would be risky for their career [55]. Participants also described negative experiences interacting with occupational services, which deterred them from accessing mental health support in the future.

All of the research participants recognised progress towards promoting and managing mental health within the medical profession. Participants described how attitudes towards mental health had improved acutely over the last 18 months, which may reflect extra efforts to support medical workers during the COVID-19 pandemic [56]. In line with previous findings [57] [58], the medical students and junior doctors noted the importance of peer support. However, a majority of participants indicated that the pandemic had limited their access to support and coping strategies which would usually benefit their wellbeing. Despite this, participants suggested that working in the medical profession had been somewhat protective for their mental wellbeing during COVID-19, in that it is an inherently helpful, rewarding and sociable profession.

To our knowledge, no qualitative studies have simultaneously examined the mental health of medical students and junior doctors during the COVID-19 pandemic. A further study strength is that an independent researcher carried out the interviews and analysis, adding an extra layer of privacy protection and strong reassurance of participant confidentiality. This study accessed a very unique cohort of medical students and junior doctors working in unprecedented times for the medical profession, therefore adding to the current sparse literature [59]. Despite this, the representativeness and transferability of the findings may be

For peer review only - http://bmjopen.bmj.com/site/about/guidelines.xhtml

limited, as most participants were males and of a white-British ethnicity. This is of particular concern as women, and ethnically diverse medics were reported to have suffered from higher rates of anxiety, depression and stress during the COVID-19 pandemic [60-62]. Additionally, whilst snowball sampling proved a useful recruitment technique, selection and response biases cannot be excluded [63], and the sample may not represent the experience of medics across the UK.

Protective measures should be implemented to minimise medical students' and junior doctors' occupational stress. These might include ensuring that workers only work their scheduled hours, encouraging more autonomy in shift allocation, and consistently monitoring their wellbeing [64]. In particular, communications should be aimed towards leadership structures, to promote supportive and compassionate cultures, and to role model setting boundaries around work-life balance [6 22]. These practices should be in place from medical school onwards, when first year students begin to be socialised into the profession [24]. Finally, individuals who need to take time off should feel able to do so and be made aware of the support systems in place [56].

The importance of peer support was clear in the findings. Consequently, interventions which foster peer support must be considered, and existing support from colleagues, supervisors and teams should be utilised [22]. Elsewhere, good practices have included 'buddying-up' schemes to reduce isolation and peer support groups where medics can discuss the emotional impact of their work [56 65]. Furthermore, the career implications of disclosing mental health problems must be clarified. Educational and training interventions may be adopted, which can encourage help seeking behaviours and boost medics' resilience [64 66 67].

For peer review only - http://bmjopen.bmj.com/site/about/guidelines.xhtml

Given the exploratory nature of this research, larger qualitative and quantitative studies are required to determine the generalisability and transferability of the findings. In particular, studies investigating perceived differences in mental health problems by sociodemographic, educational and geographical factors are warranted. Future studies may benefit from exploring the views of educational, occupational and healthcare management staff, to add other perspectives on the factors affecting this cohort's wellbeing. Further validation of the findings could also be added using observational research in order to provide more impetus for quality improvement interventions to be implemented.

As we move away from the pressures of COVID-19 pandemic, the systemic and cultural issues within medicine remain highly relevant. This paper advocates for improvement to working conditions, more communication and connection within teams, and for cultural change, in medical education and in health services.

Acknowledgements: The authors thank all the participants for sharing their experiences.

Author contributions: JT - conceived and designed the study; conducted the participant interviews, data analyses and drafted the manuscript; ED –contributed to the interpretation of the results and data analysis and with critical revisions of the manuscript.

Data statement: The data is not currently available for further use.

Ethical approval: Ethical approval was obtained from the University of Glasgow, School of Social & Political Sciences committee (PGT/SPS/2021/035/GLOB). Patients or the public were not involved in the design and conduct of this study.

Funding statement: ED acknowledges funding from the Medical Research Council (MC_UU_00022/2) and the Chief Scientist Office (SPHSU17).

For peer review only - http://bmjopen.bmj.com/site/about/guidelines.xhtml

Competing interests: An initial sample of participants were recruited through personal contacts of the lead researcher and the remainder were recruited using snowball sampling. ED declares no competing interests.

References

1. Lai CC, Shih TP, Ko WC, et al. Severe acute respiratory syndrome coronavirus 2 (SARS-CoV-2) and coronavirus disease-2019 (COVID-19): The epidemic and the challenges. *Int J Antimicrob Agents* 2020;55(3):105924. doi: 10.1016/j.ijantimicag.2020.105924 [published Online First: 2020/02/23]

2. Thome J, Deloyer J, Coogan AN, et al. The impact of the early phase of the COVID-19 pandemic on mental-health services in Europe. *World J Biol Psychiatry* 2020:1-10. doi: 10.1080/15622975.2020.1844290 [published Online First: 2020/11/05]

3. Menon A, Klein EJ, Kollars K, et al. Medical Students Are Not Essential Workers: Examining Institutional Responsibility During the COVID-19 Pandemic. *Acad Med* 2020;95(8):1149-51. doi: 10.1097/ACM.0000000000003478 [published Online First: 2020/04/30]

4. Arumi A, Bulbena-Cabre A, Bulbena A. First Person Account COVID 19 Delirium in a Doctor: When Death Stalks the Mind. *Front Psychiatry* 2021;12:626648. doi: 10.3389/fpsyt.2021.626648 [published Online First: 2021/02/16]

5. Michaeli D, Keough G, Perez-Dominguez F, et al. Medical education and mental health during COVID-19: a survey across 9 countries. *Int J Med Educ* 2022;13:35-46. doi: 10.5116/ijme.6209.10d6 [published Online First: 2022/03/01]

6. Spiers J, Buszewicz M, Chew-Graham C, et al. What challenges did junior doctors face while working during the COVID-19 pandemic? A qualitative study. *BMJ Open*

For peer review only

2021;11(12):e056122. doi: 10.1136/bmjopen-2021-056122 [published Online First: 2021/12/15]

7. Rainbow S, Dorji T. Impact of COVID-19 on medical students in the United Kingdom. *GERMS* 2020;10(3):240-43. doi: 10.18683/germs.2020.1210

8. Papapanou M, Routsi E, Tsamakis K, et al. Medical education challenges and innovations during COVID-19 pandemic. *Postgrad Med J* 2022;98(1159):321-27. doi: 10.1136/postgradmedj-2021-140032 [published Online First: 2021/03/31]

9. Ali A, Staunton M, Quinn A, et al. Exploring medical students' perceptions of the challenges and benefits of volunteering in the intensive care unit during the COVID-19 pandemic: a qualitative study. *BMJ Open* 2021;11(12):e055001. doi: 10.1136/bmjopen-2021-055001 [published Online First: 2021/12/26]

10. Harvey A. Covid-19: medical schools given powers to graduate final year students early to help NHS. *BMJ* 2020;368:m1227. doi: 10.1136/bmj.m1227 [published Online First: 2020/03/29]

11. Monrouxe LV, Bullock A, Gormley G, et al. New graduate doctors' preparedness for practice: a multistakeholder, multicentre narrative study. *BMJ Open* 2018;8(8):e023146. doi: 10.1136/bmjopen-2018-023146 [published Online First: 2018/08/31]

12. Pandey U, Corbett G, Mohan S, et al. Anxiety, Depression and Behavioural Changes in Junior Doctors and Medical Students Associated with the Coronavirus Pandemic: A Cross-Sectional Survey. *J Obstet Gynaecol India* 2020:1-5. doi: 10.1007/s13224-020-01366-w [published Online First: 2020/09/30]

13. Dunning A, Teoh K, Martin J, et al. Relationship between working conditions and psychological distress experienced by junior doctors in the UK during the COVID-19

For peer review only - http://bmjopen.bmj.com/site/about/guidelines.xhtml

pandemic: a cross-sectional survey study. *BMJ Open* 2022;12(8):e061331. doi: 10.1136/bmjopen-2022-061331 [published Online First: 2022/08/24]

14. Pappa S, Ntella V, Giannakas T, et al. Prevalence of depression, anxiety, and insomnia among healthcare workers during the COVID-19 pandemic: A systematic review and meta-analysis. *Brain Behav Immun* 2020;88:901-07. doi: 10.1016/j.bbi.2020.05.026 [published Online First: 2020/05/22]
15. Farrell SM, Kadhum M, Lewis T, et al. Wellbeing and burnout amongst medical students in England. *Int Rev Psychiatry* 2019;31(7-8):579-83. doi: 10.1080/09540261.2019.1675960 [published Online First: 2019/11/07]
16. Moir F, Yielder J, Sanson J, et al. Depression in medical students: current insights. *Adv Med Educ Pract* 2018;9:323-33. doi: 10.2147/AMEP.S137384 [published Online First: 2018/05/17]
17. Henning MA, Sollers J, Strom JM, et al. Junior doctors in their first year: mental health, quality of life, burnout and heart rate variability. *Perspect Med Educ* 2014;3(2):136-43. doi: 10.1007/s40037-013-0075-y [published Online First: 2014/04/08]
18. Bhugra D, Sauerteig SO, Bland D, et al. A descriptive study of mental health and wellbeing of doctors and medical students in the UK. *Int Rev Psychiatry* 2019;31(7-8):563-68. doi: 10.1080/09540261.2019.1648621 [published Online First: 2019/08/29]
19. Royal College of Physicians. Being a junior doctor: Experiences from the front line of the NHS. *London* 2016
20. Zeng W, Chen R, Wang X, et al. Prevalence of mental health problems among medical students in China: A meta-analysis. *Medicine (Baltimore)* 2019;98(18):e15337. doi: 10.1097/MD.0000000000015337 [published Online First: 2019/05/03]

For peer review only

21. Zivanovic R, McMillan J, Lovato C, et al. Death by Suicide among Canadian Medical Students: A National Survey-Based Study. *Can J Psychiatry* 2018;63(3):178-81. doi: 10.1177/0706743717746663 [published Online First: 2018/03/08]

22. Riley R, Buszewicz M, Kokab F, et al. Sources of work-related psychological distress experienced by UK-wide foundation and junior doctors: a qualitative study. *BMJ Open* 2021;11(6):e043521. doi: 10.1136/bmjopen-2020-043521 [published Online First: 2021/06/25]

23. Riley R, Spiers J, Buszewicz M, et al. What are the sources of stress and distress for general practitioners working in England? A qualitative study. *BMJ Open* 2018;8(1):e017361. doi: 10.1136/bmjopen-2017-017361 [published Online First: 2018/01/13]

24. Wong AMF. Beyond burnout: looking deeply into physician distress. *Can J Ophthalmol* 2020;55(3 Suppl 1):7-16. doi: 10.1016/j.jcjo.2020.01.014 [published Online First: 2020/03/25]

25. Lock FK, Carrieri D. Factors affecting the UK junior doctor workforce retention crisis: an integrative review. *BMJ Open* 2022;12(3):e059397. doi: 10.1136/bmjopen-2021-059397 [published Online First: 2022/03/31]

26. Zis P, Artemiadis A, Bargiotas P, et al. Medical Studies during the COVID-19 Pandemic: The Impact of Digital Learning on Medical Students' Burnout and Mental Health. *Int J Environ Res Public Health* 2021;18(1) doi: 10.3390/ijerph18010349 [published Online First: 2021/01/21]

27. Essadek A, Gressier F, Robin M, et al. Mental health of medical students during the COVID19: Impact of studies years. *J Affect Disord Rep* 2022;8:100318. doi: 10.1016/j.jadr.2022.100318 [published Online First: 2022/02/16]

For peer review only - http://bmjopen.bmj.com/site/about/guidelines.xhtml

28. O'Byrne L, Gavin B, Adamis D, et al. Levels of stress in medical students due to COVID-19. *J Med Ethics* 2021 doi: 10.1136/medethics-2020-107155 [published Online First: 2021/03/05]

29. Warren J, Plunkett E, Rudge J, et al. Trainee doctors' experiences of learning and well-being while working in intensive care during the COVID-19 pandemic: a qualitative study using appreciative inquiry. *BMJ Open* 2021;11(5):e049437. doi: 10.1136/bmjopen-2021-049437 [published Online First: 2021/05/27]

30. Byrne JP, Creese J, Matthews A, et al. '...the way it was staffed during COVID is the way it should be staffed in real life...': a qualitative study of the impact of COVID-19 on the working conditions of junior hospital doctors. *BMJ Open* 2021;11(8):e050358. doi: 10.1136/bmjopen-2021-050358 [published Online First: 2021/08/11]

31. Olah B, Radi BM, Kosa K. Barriers to Seeking Mental Help and Interventions to Remove Them in Medical School during the COVID-19 Pandemic: Perspectives of Students. *Int J Environ Res Public Health* 2022;19(13) doi: 10.3390/ijerph19137662 [published Online First: 2022/07/10]

32. Kromydas T, Green M, Craig P, et al. Comparing population-level mental health of UK workers before and during the COVID-19 pandemic: a longitudinal study using Understanding Society. *Journal of Epidemiology and Community Health* 2022;76(6):527. doi: 10.1136/jech-2021-218561

33. Mutambudzi M, Niedwiedz C, Macdonald EB, et al. Occupation and risk of severe COVID-19: prospective cohort study of 120 075 UK Biobank participants. *Occupational and Environmental Medicine* 2020

34. Griffin L, Riley R. Exploring the psychological impact of working during COVID-19 on medical and nursing students: a qualitative study. *BMJ Open* 2022;12(6):e055804. doi: 10.1136/bmjopen-2021-055804 [published Online First: 2022/06/24]

For peer review only - http://bmjopen.bmj.com/site/about/guidelines.xhtml

35. Wilkinson C, Finn G, Crampton P. Responsibility with a Safety Net: Exploring the Medical Student to Junior Doctor Transition During COVID-19. *Medical Science Educator* 2021;32(1):121-29. doi: 10.1007/s40670-021-01476-8

36. Petrino R, Riesgo LG, Yilmaz B. Burnout in emergency medicine professionals after 2 years of the COVID-19 pandemic: a threat to the healthcare system? *Eur J Emerg Med* 2022;29(4):279-84. doi: 10.1097/MEJ.0000000000000952 [published Online First: 2022/05/28]

37. Zhou T, Xu C, Wang C, et al. Burnout and well-being of healthcare workers in the post-pandemic period of COVID-19: a perspective from the job demands-resources model. *BMC Health Serv Res* 2022;22(1):284. doi: 10.1186/s12913-022-07608-z [published Online First: 2022/03/04]

38. Palinkas LA, Horwitz SM, Green CA, et al. Purposeful Sampling for Qualitative Data Collection and Analysis in Mixed Method Implementation Research. *Adm Policy Ment Health* 2015;42(5):533-44. doi: 10.1007/s10488-013-0528-y [published Online First: 2013/11/07]

39. Noy C. Sampling Knowledge: The Hermeneutics of Snowball Sampling in Qualitative Research. *International Journal of Social Research Methodology* 2008;11(4):327-44. doi: 10.1080/13645570701401305

40. Naderifar M, Goli H, Ghaljaie F. Snowball Sampling: A Purposeful Method of Sampling in Qualitative Research. *Strides in Development of Medical Education* 2017;14(3) doi: 10.5812/sdme.67670

41. Sanjari M, Bahramnezhad F, Fomani FK, et al. Ethical challenges of researchers in qualitative studies: the necessity to develop a specific guideline. *J Med Ethics Hist Med* 2014;7:14. [published Online First: 2014/12/17]

For peer review only

42. Antoni A, Beer H. Research Impact as Care: Re-Conceptualizing Research Impact From an Ethics of Care Perspective. *Business Ethics and Care in Organizations* 2019

43. Schofield G, Dittborn M, Selman LE, et al. Defining ethical challenge(s) in healthcare research: a rapid review. *BMC Med Ethics* 2021;22(1):135. doi: 10.1186/s12910-021-00700-9 [published Online First: 2021/10/01]

44. Williamson AE, Burns N. The safety of researchers and participants in primary care qualitative research. *Br J Gen Pract* 2014;64(621):198-200. doi: 10.3399/bjgp14X679480 [published Online First: 2014/04/02]

45. Keen S, Lomeli-Rodriguez M, Joffe H. From Challenge to Opportunity: Virtual Qualitative Research During COVID-19 and Beyond. *Int J Qual Methods* 2022;21:16094069221105075. doi: 10.1177/16094069221105075 [published Online First: 2022/06/14]

46. O'Brien BC, Harris IB, Beckman TJ, et al. Standards for Reporting Qualitative Research: A Synthesis of Recommendations. *Academic Medicine* 2014;89(9)

47. Braun V, Clarke V. Reflecting on reflexive thematic analysis. *Qualitative Research in Sport, Exercise and Health* 2019;11(4)

48. Palinkas LA. Qualitative and mixed methods in mental health services and implementation research. *J Clin Child Adolesc Psychol* 2014;43(6):851-61. doi: 10.1080/15374416.2014.910791 [published Online First: 2014/10/29]

49. Wainwright E, Fox F, Breffni T, et al. Coming back from the edge: a qualitative study of a professional support unit for junior doctors. *BMC Med Educ* 2017;17(1):142. doi: 10.1186/s12909-017-0978-0 [published Online First: 2017/08/25]

50. Smith SE, Tallentire VR, Pope LM, et al. Foundation Year 2 doctors' reasons for leaving UK medicine: an in-depth analysis of decision-making using semistructured

For peer review only - http://bmjopen.bmj.com/site/about/guidelines.xhtml

interviews. *BMJ Open* 2018;8(3):e019456. doi: 10.1136/bmjopen-2017-019456 [published Online First: 2018/03/04]

51. Creese J, Byrne JP, Conway E, et al. "We All Really Need to just Take a Breath": Composite Narratives of Hospital Doctors' Well-Being during the COVID-19 Pandemic. *Int J Environ Res Public Health* 2021;18(4) doi: 10.3390/ijerph18042051 [published Online First: 2021/03/07]

52. Henderson M, Brooks SK, del Busso L, et al. Shame! Self-stigmatisation as an obstacle to sick doctors returning to work: a qualitative study: Table 1. *BMJ Open* 2012;2(5) doi: 10.1136/bmjopen-2012-001776

53. Bianchi EF, Bhattacharyya MR, Meakin R. Exploring senior doctors' beliefs and attitudes regarding mental illness within the medical profession: a qualitative study. *BMJ Open* 2016;6(9):e012598. doi: 10.1136/bmjopen-2016-012598 [published Online First: 2016/09/18]

54. Waugh W, Lethem C, Sherring S, et al. Exploring experiences of and attitudes towards mental illness and disclosure amongst health care professionals: a qualitative study. *J Ment Health* 2017;26(5):457-63. doi: 10.1080/09638237.2017.1322184 [published Online First: 2017/05/11]

55. Schwenk TL, Davis L, Wimsatt LA. Depression, stigma, and suicidal ideation in medical students. *JAMA* 2010;304(11):1181-90. doi: 10.1001/jama.2010.1300 [published Online First: 2010/09/16]

56. BMA. The mental health and wellbeing of the medical workforce – now and beyond COVID-19. 2021

57. Fisher EB, Ayala GX, Ibarra L, et al. Contributions of Peer Support to Health, Health Care, and Prevention: Papers from Peers for Progress. *Ann Fam Med* 2015;13 Suppl 1:S2-8. doi: 10.1370/afm.1852 [published Online First: 2015/08/26]

For peer review only - http://bmjopen.bmj.com/site/about/guidelines.xhtml

58. Moir F, Henning M, Hassed C, et al. A Peer-Support and Mindfulness Program to Improve the Mental Health of Medical Students. *Teach Learn Med* 2016;28(3):293-302. doi: 10.1080/10401334.2016.1153475 [published Online First: 2016/04/20]

59. Kerins J, Hamilton AL, Pringle J, et al. Exploring the impact of the COVID-19 pandemic on doctors' core workplace needs: a qualitative study of internal medicine trainees in Scotland. *BMJ Open* 2021;11(6):e053506. doi: 10.1136/bmjopen-2021-053506 [published Online First: 2021/07/02]

60. Morgan R, Tan HL, Oveisi N, et al. Women healthcare workers' experiences during COVID-19 and other crises: A scoping review. *Int J Nurs Stud Adv* 2022;4:100066. doi: 10.1016/j.ijnsa.2022.100066 [published Online First: 2022/02/08]

61. Regenold N, Vindrola-Padros C. Gender Matters: A Gender Analysis of Healthcare Workers' Experiences during the First COVID-19 Pandemic Peak in England. *Social Sciences* 2021;10(2) doi: 10.3390/socsci10020043

62. Qureshi I, Gogoi M, Wobi F, et al. Healthcare Workers From Diverse Ethnicities and Their Perceptions of Risk and Experiences of Risk Management During the COVID-19 Pandemic: Qualitative Insights From the United Kingdom-REACH Study. *Frontiers in Medicine* 2022;9 doi: 10.3389/fmed.2022.930904

63. Pannucci CJ, Wilkins EG. Identifying and avoiding bias in research. *Plast Reconstr Surg* 2010;126(2):619-25. doi: 10.1097/PRS.0b013e3181de24bc [published Online First: 2010/08/04]

64. Vizheh M, Qorbani M, Arzaghi SM, et al. The mental health of healthcare workers in the COVID-19 pandemic: A systematic review. *J Diabetes Metab Disord* 2020:1-12. doi: 10.1007/s40200-020-00643-9 [published Online First: 2020/11/03]

For peer review only - http://bmjopen.bmj.com/site/about/guidelines.xhtml

65. Behrman S, Baruch N, Stegen G. Peer support for junior doctors: a positive outcome of the COVID-19 pandemic? *Future Healthc J* 2020;7(3):e64-e66. doi: 10.7861/fhj.2020-0069 [published Online First: 2020/10/24]

66. Yiu V. Supporting the well-being of medical students. *CMAJ* 2005;172(7):889-90. doi: 10.1503/cmaj.050126 [published Online First: 2005/03/30]

67. Steen M, Othman SME, Briley A, et al. Self-compassion Education for Health Professionals (Nurses and Midwives): Protocol for a Sequential Explanatory Mixed Methods Study. *JMIR Res Protoc* 2022;11(1):e34372. doi: 10.2196/34372 [published Online First: 2021/12/02]

Figure Legends

Figure 1**.** Thematic Map

For peer review only

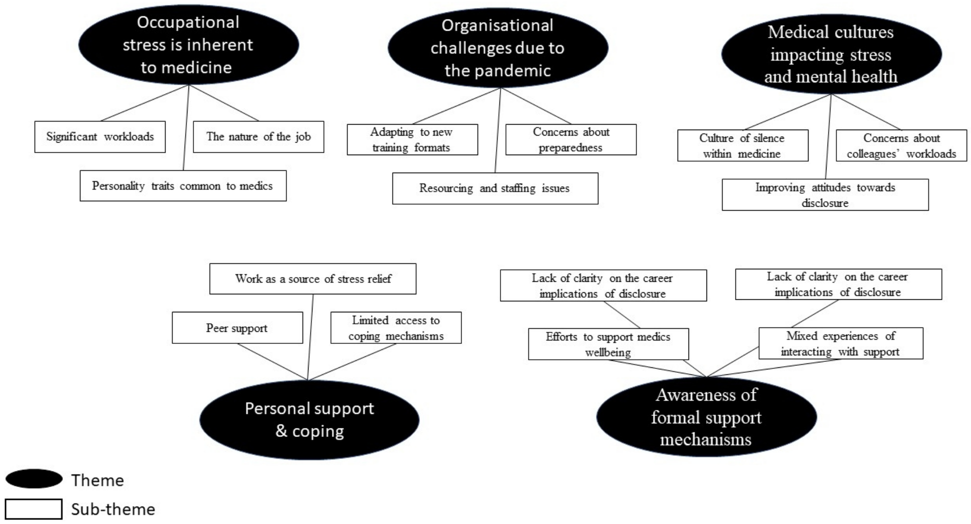

Figure 1. Thematic Map

338x190mm (96 x 96 DPI)

Interview Guide

A qualitative study to explore UK medical students' and junior doctors' experiences of occupational stress and mental health during the COVID-19 pandemic.

Warm up questions

How are you today?

How has work/university been recently?

Have you got any plans this summer?

Main Questions

Experiences of occupational stress

Can you describe a time when you have experienced work-related stress?

Do you make any conscious efforts to reduce work-related stress?

Have you ever felt as though work-related stress has had an impact on your mental wellbeing?

Do you feel as though you are under more pressure than other occupations/degree subjects?

How has the pandemic influenced your experiences of stress/ work stress?

Has the pandemic limited your ability to manage your stress?

Is there anything that you think has been helping your mental health and wellbeing during the pandemic?

Is there anything you have done to try to relieve stress during the pandemic?

Attitudes towards mental illness

Do you feel as though working in the health profession increases or reduces your risk of suffering from a mental illness? – Discuss risk factors and protective factors

Would you agree with the perception that only certain people suffer from mental illnesses? If so, what type of person? Any particular personality traits/ behaviours

How do you feel colleagues would view a co-worker taking leave for mental ill health compared to a physical condition? Why may this be the case?

For peer review only

Barriers to disclosure

Hypothetically if you were suffering from mentally ill health, would there be any factors that may prevent you from disclosing it?

Would you be concerned at all about job stability if you were disclosing a mental illness to a manager or colleagues?

Would you be concerned about experiencing stigma from co-workers if you disclosed a mental illness?

If you were suffering from a mental illness what support networks would be available for you? What would be your preferred route?

Would access to help services be limited by the COVID pandemic?

Cool down questions

Did you find everything okay with the interview?

Do you have any questions or anything you'd like to clarify?

Are you doing anything after this?

Thank participants for their time and for meeting with me.

For peer review only

Standards for Reporting Qualitative Research (SRQR)*

http://www.equator-network.org/reporting-guidelines/srqr/

Title and abstract

	Page/line no(s).
**Title** - Concise description of the nature and topic of the study Identifying the study as qualitative or indicating the approach (e.g., ethnography, grounded theory) or data collection methods (e.g., interview, focus group) is recommended	Page 2, line 3
**Abstract** - Summary of key elements of the study using the abstract format of the intended publication; typically includes background, purpose, methods, results, and conclusions	Page 3, line 3

Introduction

**Problem formulation** - Description and significance of the problem/phenomenon studied; review of relevant theory and empirical work; problem statement	Page 5, Line 5
**Purpose or research question** - Purpose of the study and specific objectives or questions	Page 7 Line 17

Methods

**Qualitative approach and research paradigm** - Qualitative approach (e.g., ethnography, grounded theory, case study, phenomenology, narrative research) and guiding theory if appropriate; identifying the research paradigm (e.g., postpositivist, constructivist/ interpretivist) is also recommended; rationale**	Page 8, line 54
**Researcher characteristics and reflexivity** - Researchers' characteristics that may influence the research, including personal attributes, qualifications/experience, relationship with participants, assumptions, and/or presuppositions; potential or actual interaction between researchers' characteristics and the research questions, approach, methods, results, and/or transferability	Page 7, line 51
**Context** - Setting/site and salient contextual factors; rationale**	Page 8, line 4
**Sampling strategy** - How and why research participants, documents, or events were selected; criteria for deciding when no further sampling was necessary (e.g., sampling saturation); rationale**	Page 7, line 49
**Ethical issues pertaining to human subjects** - Documentation of approval by an appropriate ethics review board and participant consent, or explanation for lack thereof; other confidentiality and data security issues	Page 8, line 6
**Data collection methods** - Types of data collected; details of data collection procedures including (as appropriate) start and stop dates of data collection and analysis, iterative process, triangulation of sources/methods, and modification of procedures in response to evolving study findings; rationale**	Page 8, line 24

For peer review only - http://bmjopen.bmj.com/site/about/guidelines.xhtml

**Data collection instruments and technologies** - Description of instruments (e.g., interview guides, questionnaires) and devices (e.g., audio recorders) used for data collection; if/how the instrument(s) changed over the course of the study	Page 8, line 29
**Units of study** - Number and relevant characteristics of participants, documents, or events included in the study; level of participation (could be reported in results)	Page 9, line 18
**Data processing** - Methods for processing data prior to and during analysis, including transcription, data entry, data management and security, verification of data integrity, data coding, and anonymization/de-identification of excerpts	Page 8, line 29
**Data analysis** - Process by which inferences, themes, etc., were identified and developed, including the researchers involved in data analysis; usually references a specific paradigm or approach; rationale**	Page 8, line 54
**Techniques to enhance trustworthiness** - Techniques to enhance trustworthiness and credibility of data analysis (e.g., member checking, audit trail, triangulation); rationale**	Page 9, line 3

Results/findings

**Synthesis and interpretation** - Main findings (e.g., interpretations, inferences, and themes); might include development of a theory or model, or integration with prior research or theory	Page 11, line 10
**Links to empirical data** - Evidence (e.g., quotes, field notes, text excerpts, photographs) to substantiate analytic findings	Page 11, Line 24

Discussion

**Integration with prior work, implications, transferability, and contribution(s) to the field** - Short summary of main findings; explanation of how findings and conclusions connect to, support, elaborate on, or challenge conclusions of earlier scholarship; discussion of scope of application/generalizability; identification of unique contribution(s) to scholarship in a discipline or field	Page 20, Line 4
**Limitations** - Trustworthiness and limitations of findings	Page 22, Line 1

Other

**Conflicts of interest** - Potential sources of influence or perceived influence on study conduct and conclusions; how these were managed	Page 24, Line 4
**Funding** - Sources of funding and other support; role of funders in data collection, interpretation, and reporting	Page 23, Line 56

*The authors created the SRQR by searching the literature to identify guidelines, reporting standards, and critical appraisal criteria for qualitative research; reviewing the reference lists of retrieved sources; and contacting experts to gain feedback. The SRQR aims to improve the transparency of all aspects of qualitative research by providing clear standards for reporting qualitative research.

For peer review only

**The rationale should briefly discuss the justification for choosing that theory, approach, method, or technique rather than other options available, the assumptions and limitations implicit in those choices, and how those choices influence study conclusions and transferability. As appropriate, the rationale for several items might be discussed together.

Reference:

O'Brien BC, Harris IB, Beckman TJ, Reed DA, Cook DA. **Standards for reporting qualitative research: a synthesis of recommendations.** *Academic Medicine*, Vol. 89, No. 9 / Sept 2014 DOI: 10.1097/ACM.0000000000000388

For peer review only

BMJ Open

A qualitative study to explore UK medical students' and junior doctors' experiences of occupational stress and mental health during the COVID-19 pandemic

Journal:	*BMJ Open*
Manuscript ID	bmjopen-2022-065639.R2
Article Type:	Original research
Date Submitted by the Author:	14-Nov-2022
Complete List of Authors:	Tawse, James; University of Glasgow College of Science and Engineering; Alder Hey Children's Hospital<br>Demou, Evangelia; University of Glasgow College of Medical Veterinary and Life Sciences, MRC/CSO Social and Public Health Sciences Unit
**Primary Subject Heading**:	Mental health
Secondary Subject Heading:	Occupational and environmental medicine, Public health, Qualitative research, Medical education and training
Keywords:	MENTAL HEALTH, COVID-19, MEDICAL EDUCATION & TRAINING, OCCUPATIONAL & INDUSTRIAL MEDICINE, Health & safety < HEALTH SERVICES ADMINISTRATION & MANAGEMENT

SCHOLARONE™
Manuscripts

I, the Submitting Author has the right to grant and does grant on behalf of all authors of the Work (as defined in the below author licence), an exclusive licence and/or a non-exclusive licence for contributions from authors who are: i) UK Crown employees; ii) where BMJ has agreed a CC-BY licence shall apply, and/or iii) in accordance with the terms applicable for US Federal Government officers or employees acting as part of their official duties; on a worldwide, perpetual, irrevocable, royalty-free basis to BMJ Publishing Group Ltd ("BMJ") its licensees and where the relevant Journal is co-owned by BMJ to the co-owners of the Journal, to publish the Work in this journal and any other BMJ products and to exploit all rights, as set out in our licence.

The Submitting Author accepts and understands that any supply made under these terms is made by BMJ to the Submitting Author unless you are acting as an employee on behalf of your employer or a postgraduate student of an affiliated institution which is paying any applicable article publishing charge ("APC") for Open Access articles. Where the Submitting Author wishes to make the Work available on an Open Access basis (and intends to pay the relevant APC), the terms of reuse of such Open Access shall be governed by a Creative Commons licence – details of these licences and which Creative Commons licence will apply to this Work are set out in our licence referred to above.

Other than as permitted in any relevant BMJ Author's Self Archiving Policies, I confirm this Work has not been accepted for publication elsewhere, is not being considered for publication elsewhere and does not duplicate material already published. I confirm all authors consent to publication of this Work and authorise the granting of this licence.

For peer review only

A qualitative study to explore UK medical students' and junior doctors' experiences of occupational stress and mental health during the COVID-19 pandemic

James Tawse[1,2*], Evangelia Demou[3]

1. College of Social Sciences, University of Glasgow, Glasgow, UK

2. Alder Hey Children's Hospital, Liverpool, UK

3. MRC/CSO Social and Public Health Sciences Unit, Institute of Health and Wellbeing, University of Glasgow, Glasgow, UK

Corresponding author:
Mr James Tawse
Alder Hey Children's Hospital,
Liverpool,
UK
Email: james.tawse@alderhey.nhs.uk

For peer review only

Word count: 4,207

Abstract

Objectives: This qualitative study aimed to explore the occupational experiences of medical students and junior doctors working during the COVID-19 pandemic. In particular, the research sought to identify factors which mediated work stress; barriers to disclosing mental health problems; and levels of support medical students and junior doctors received during the pandemic.

Design: This study was a form of thematic analysis and adopted an inductive, 'bottom-up' approach, in which coded categories were derived from rich, descriptive data.

Setting: Semi-structured interviews were conducted online with UK based medical students and junior doctors. Interviews were recorded, and analysis was done by coding salient quotes into themes.

Participants: The final sample consisted of 7 junior doctors and 8 medical students, during the summer of 2021.

Results: High levels of occupational stress were identified which were exacerbated by COVID-19. A number of organisational difficulties associated with the pandemic compounded participants' experiences of work stress. Participants recognised progress toward promoting and managing mental health within the profession but may still be reluctant to access support services. Barriers to disclosure included fear of stigmatisation, concerns about adding to colleagues' workloads, lack of clarity about career implications and mistrust of occupational health services.

Conclusions: Whilst attitudes towards mental health have improved, medical students and junior doctors may avoid seeking help. Given the immense pressures faced by health services, it is imperative that extra measures are implemented to minimise work-stress, encourage help-seeking behaviours and promote supportive work cultures.

For peer review only - http://bmjopen.bmj.com/site/about/guidelines.xhtml

Article Summary

Strengths and limitations of this study:

- This research captured medical students and junior doctors' experiences of working during the summer of 2021; alongside rising rates of COVID in the UK, ongoing restrictions to social contact and unprecedented pressure on health services.
- This study accessed a unique cohort of medical students and junior doctors, who did not receive standard training opportunities.
- Thematic analysis was conducted on the interview data, facilitating the development of comprehensive and insightful themes.
- The representativeness and transferability of the findings may be limited, as most participants were males and of a white-British ethnicity.

Keywords: medical student, junior doctor, mental health, occupational stress, COVID-19

For peer review only

Introduction

Coronavirus disease (COVID-19) is one of the most severe international health problems in the last decades [1]. Communities worldwide suffered directly from high infection rates associated with increased mortality, and indirectly through measures to contain the spreading of the virus [2]. In the UK, education, workplaces and health systems had to radically adjust their working practices to adhere to social distancing, safety, and health guidelines[3 4]. For medical students and junior doctors, the pandemic presented an unexpected, disruptive external shock which created uncertainty and distress [5 6].

Since the onset of the pandemic, medical students experienced drastic changes to their academic curricula [7]. Most medical schools shifted to online learning with a reduction or even suspension of practical teaching [5]. Medical students were faced with the discontinuation of clinical placements, novel examination formats and studying in isolation [8]. In response to the public health crisis, medical students across the UK volunteered in hospitals and General Practices, to support shortages in medical staff [7]. However, this placed volunteers at an increased risk of infection, and presented challenges to provide students with adequate supervision [9]. Furthermore, UK governing bodies allowed final year medical students to graduate 6-8 weeks early from medical school to join the frontline in fighting against the pandemic [10]. This raises questions about this group of newly graduated doctors' preparedness to enter the profession, under difficult circumstances, and is concerning given that less prepared trainees make more medical errors and suffer from higher rates of burnout [11].

Junior doctors working to fight COVID-19 reported stress and distress due to the strain on healthcare systems [6]. Such stressors included understaffing, increased workload and uncertainty due to major changes to working practices [12 13]. On top of this, healthcare

For peer review only - http://bmjopen.bmj.com/site/about/guidelines.xhtml

professionals' resilience was hindered by loss of support, infection of friends and family, as well as exposure to unprecedented levels of suffering and illness, both of colleagues and patients[14].

Before the pandemic, concerns had already been raised about medical students and junior doctors' wellbeing [15-18]. Research had consistently shown that medical students and junior doctors experience significant levels of work-stress and burnout, as well as high rates of mental health problems and suicide when compared to the general population [18-21].
In recent times, systemic issues within healthcare have compounded workers' mental health difficulties [22-24]. Poor working conditions, inadequate support mechanisms, and a lack of flexibility have contributed towards record numbers of trainee doctors leaving the profession altogether [25].

The mental health burden of junior doctors and medical students working during the pandemic has been the subject of interest in quantitative [12 13 26-28] and qualitative research studies [6 13 29-34]. However, to the author's knowledge, no qualitative studies have simultaneously examined the mental health of medical students and junior doctors during COVID-19. As such, this paper can offer valuable insights into these groups' relative stress, mental wellbeing and access to support during this difficult transitionary period from student to doctor [35], and in unprecedented circumstances. This qualitative study captures the voices of these groups at a distinct and testing time, amongst rising rates of COVID-19 in the summer of 2021 and the long-term impact of COVID-19 on healthcare workers' wellbeing remains unclear [36 37]. Consequently, continued efforts to protect medical students' and junior doctors' wellbeing are warranted. In doing so, retention issues may be addressed, which threaten patient safety, and the future of health services [25].

For peer review only - http://bmjopen.bmj.com/site/about/guidelines.xhtml

This study aimed to explore medical students' and junior doctors' experiences of occupational stress and mental health during the COVID-19 pandemic in the UK. Specifically, this study addressed the following three research questions. The research questions informed the study and the interview guide (Appendix 1), they were not directly asked to the participants.

RQ1: What are the key sources of occupational stress amongst medical students and junior doctors working during the COVID-19 pandemic?

RQ2: What barriers exist to accessing mental health support within this workforce?

RQ3: Have medical students and junior doctors received sufficient support to manage their work stress and mental health challenges during the COVID-19 pandemic?

Methods

Participant recruitment

The inclusion criteria for the study were medical students and junior doctors working in the UK, during the summer of 2021. For the present study, 'junior doctor' refers to a doctor who graduated from medical school in the last five years, and who's currently enrolled on a medical training programme. Medical students were only invited to take part if they were in 4th year onwards. This cut-off was selected as these students have experience of clinical placement, and they faced significant disruption to their training, in close proximity to qualification. A combination of sampling strategies were used to recruit participants [38]. Initially, purposeful sampling of participants was implemented, where the lead researcher reached out to personal contacts and acquaintances who may have been available and willing to participate. Following this, participants were recruited using snowball sampling [39 40]. We aimed to conduct approximately 15-20 interviews.

For peer review only - http://bmjopen.bmj.com/site/about/guidelines.xhtml

Once sampled, participants were contacted to arrange one-to-one interviews and interviews were arranged for a time that suited the participants [6]. Before starting the interview, participants were made aware of the requirements and purposes of the research, the identity of the researcher, and how the results would be used [41 42]. Participants were reminded of their ethical rights surrounding confidentiality, data protection and their right to withdraw at any point [43]. A safety plan was in place, to ensure that support could be provided in the event of a concerning disclosure [6 44]. Following this, participants made an informed decision on their participation.

Semi-structured, one-on-one interviews were conducted online. The research questions were used as guides to develop the interview schedule (Appendix 1). Interviews took place over Zoom™, to adhere to the social distancing guidelines in place (July-August 2021) [45]. Recordings of interviews were taken using the built-in tool on Zoom™, for later verbatim transcription. After transcription, the recordings were erased. Interviews lasted an average of 36 minutes (range: 25 to 52 minutes).

The Standards for Reporting Qualitative Research reporting guidelines were utilised (Appendix 2) [46]. Ethical approval was obtained from the University of Glasgow, School of Social & Political Sciences Committee (PGT/SPS/2021/035/GLOB).

Patient and public involvement

Patients or the public were not involved in the design and conduct of this study.

Data analysis methodology

For peer review only - http://bmjopen.bmj.com/site/about/guidelines.xhtml

For peer review only

An inductively driven thematic analysis of the interview data was undertaken, in accordance with Braun and Clarke's six-stage model [47]. The dependability of the findings was assured by conducting regular meetings with the second researcher (ED) to produce a coding framework that accurately captured the emerging themes. Data analysis took place in parallel with interviews, which meant that the researcher could closely follow emerging themes and confirm when no new themes were arising [48]. *NVivo 12* was used to analyse the data.

Results

Participant demographic information and the thematic map are presented in Table 1 and Figure 1, respectively. The final sample consisted of 15 participants (8 medical students, 7 junior doctors). Of the medical students, 3 were in their 4th year of study, whilst 5 were in their 5th year of study. With regards to the junior doctors, 5 were in their first year of postgraduate work (FY1), whilst 2 were in their 3rd year (FY3). The participants were working across the UK; 12 were male and the majority (13/15) were of a White-British ethnic background (Table 1).

For peer review only

Table 1: Sample characteristics				
**Participant ID**	**Training level**	**Place of work/study**	**Gender Identity**	**Ethnicity**
**#1**	5th year medical student	North-West England	Male	White-British
**#2**	5th year medical student	North-West England	Male	White-British
**#3**	4th year medical student	North-East England	Male	White-British
**#4**	FY1	Scotland	Male	White-British
**#5**	FY1	Midlands England	Male	White-British
**#6**	FY1	Midlands England	Female	White-British
**#7**	5th year medical student	North-West England	Male	White-British
**#8**	4th year medical student	South-East England	Male	White-British
**#9**	FY1	Scotland	Male	White-British
**#10**	FY3	North-East England	Female	White-British
**#11**	FY1	North-West England	Male	Asian-British
**#12**	5th year medical student	North-West England	Male	White-British
**#13**	5th year medical student	Scotland	Male	White-British
**#14**	4th year medical student	Scotland	Male	White-British
**#15**	FY3	South-East England	Female	Asian-British

For peer review only - http://bmjopen.bmj.com/site/about/guidelines.xhtml

[Insert Figure 1]

Occupational stress inherent to medicine

Sizeable workloads in medicine were a significant stressor amongst participants and many reported an increased prevalence of symptoms of depression and anxiety in the build-up to exams. Some participants felt that their work commitments were more excessive than other degree subjects or occupational roles, and that this might make them more susceptible to poor mental health.

> *"I lived with non-medics this year and I was quite surprised by how little they did (...), I think when you have to turn up to placement 9-5 every day... I think that pressure drains you and you do get worn down by it" (#3, 5th year student).*

All participants stated that practising medicine can be emotionally challenging, as reflected below from a final year medical student.

> *"When you're looking at the clinical side, I do think it is particularly challenging, for example when you're put in situations where you're watching someone die or trying to save someone's life" (#2, 5th year student).*

Moreover, participants remarked that personality traits common to medics, such as competitiveness, perfectionism, self-criticism and a tendency toward workaholism, can be maladaptive in terms of their wellbeing.

For peer review only

"The people that medicine attracts can be perfectionists where they might be more vulnerable to negative experiences and working in medicine comes with its fair share of those" (#7, 4th year student).

Participants explained that you have to be resilient to practise medicine, to cope with the workload and the emotional aspects of the job. In some cases, participants alluded to a socialisation process which begins upon entry to medical school, where students must claim or develop the resilience that the profession requires.

"I do think we are quite a resilient group of people, because you kind of have to be, and it's something that just happens to you when you're in medical school and you start working" (#4, FY1).

Organisational challenges associated with the COVID-19 pandemic

A majority of medical students suggested that changes to academic structure had compounded aspects of their education as well as their mental burden during the pandemic. Participants explained how online learning mechanisms provided fewer opportunities for social interaction, led to feelings of frustration and decreased their motivation to study.

"I much prefer face-to-face because there's much more interaction and it's so much easier to engage, and so much more sociable. You kind of lose motivation when you're just staring at a screen all day in a room by yourself" (#2, 5th year student).

For peer review only - http://bmjopen.bmj.com/site/about/guidelines.xhtml

Concerns around preparedness were described, specifically where aspects of education had been impaired or missed altogether. This point is particularly salient for the sub-cohort of junior doctors who graduated early to offer their services during the pandemic.

> *"I've still never worked in the Emergency Department (ED), I've never even been there as a medical student, so when it eventually comes to me being in ED, I'll definitely feel less prepared in that regard" (#5, FY1).*

Despite this, several junior doctors suggested that even without the pandemic, they would have felt underprepared for the step up from medical school to a qualified doctor.

> *"You get to the point in medical school where you've done your exams and you're going to be as ready as you will be" (#4, FY1).*

Another source of stress, which particularly pertained to junior doctors, was resourcing problems within healthcare. For example, staffing problems left junior doctors feeling overburdened which compounded their experiences of work stress and impacted upon the quality of care they could provide.

> *"The consultant came back and kind of said... you know, 'Have you done this? Have you done this? Has this happened?' and I was just oblivious to it. It was a particularly understaffed ward in a time when the hospital was understaffed" (#4, FY1).*

In some cases, a shortage of personal protective equipment (PPE) led to concerns around risk of infection and personal health and safety. Moreover, participants suggested that a lack of

For peer review only

resources including ventilators and hospital beds had created intensely stressful situations, where staff would regularly be making life and death decisions about who should receive care.

> *"That's quite hard knowing that you're sort of holding off optimal treatment for a patient because you don't have beds. Yeah, that was horrible actually, because then equally you've then got families, saying 'well, why aren't we escalating?'" (#6, FY1).*

Medical cultures surrounding work-stress and mental health

Participants discussed cultural norms regarding occupational stress and mental health within the health service. A culture where healthcare workers' schedules and work-life balance were not prioritised was noted. Junior doctors explained that it was common to be asked to stay late or work on your day off at short notice. Participants described experiencing pressure and guilt when being asked to work extra hours and suggested that it was particularly difficult to decline requests during the pandemic.

> *"It's a really difficult one because you just feel like you should, you've got an obligation to do it, and it kind of became the attitude during the pandemic that you weren't doing anything anyway" (#4, FY1).*

In some cases, participants suggested that medical cultures can block access to mental health services and can perpetuate presenteeism behaviours. For example, some participants felt that it may be particularly difficult to disclose a mental health problem whilst practising medicine, due to stigmatisation. Specifically, concerns were raised that peers, tutors or supervisors may

For peer review only - http://bmjopen.bmj.com/site/about/guidelines.xhtml

perceive them as being mentally weak, or lacking the resilient personality required to practice medicine, if they were to take time off for mental health difficulties.

> *"Anyone that works does this, but particularly people that work in the NHS, you feel you have a duty to go to work, you know even if you're at death's door you go into work" (#4, FY1).*

> *".... I was worried that you know, if they do think that I'm not mentally 100% then I can't be doing this, because I can't handle the stress" (#13, 4th year student).*

All of the participants stated that attitudes towards mental health and disclosure have improved in recent times and this reflected broader perceptions and public awareness of the importance of good mental health. Moreover, participants agreed that recognition of mental health had acutely improved during the pandemic, as evidenced below.

> *"The general consensus is that the kind of historic or traditional view of that is kind of 'pick yourself and get on with it', whereas I think over the last 12 months, but also over the last decade, there's definitely been a shift in acknowledging that health care professionals need to take care of themselves first and foremost" (#1 , 5th year student).*

A number of participants indicated that they would not feel comfortable approaching senior doctors within their hospitals for mental health support. Despite this, interactions with senior doctors were generally positive and participants felt that they were helpful and accessible.

For peer review only - http://bmjopen.bmj.com/site/about/guidelines.xhtml

"All through medical school you're taught about these really scary surgeons and consultants that won't take your answers but, on the whole, they've all been really supportive" (#6, FY1).

Junior doctors worried about burdening colleagues with extra work if they were to take time off, mainly due to significant staff shortages during the pandemic. Despite this, medical students and junior doctors adopted an altruistic perspective, whereby they would feel guilty for adding to their colleagues' workloads but noted that they would be happy to pick up the extra work if their colleagues needed time off for their mental wellbeing.

"That's the good thing about being an FY1, you have such good camaraderie, you know you would do that (pick up the extra work when a colleague is off) for your colleagues, there's a real sense of that amongst the workforce" (#4, FY1).

Personal support & coping during the pandemic

The importance of peer support was unanimously felt by all participants. Many relied on their peers for informal mental health support and to problem solve difficult situations. A majority of the participants also lived with their course-mates or colleagues, which provided them with additional opportunities to open up about their problems.

"We kind of do it without being labelled a support network, so we all come home from placement and will be like, 'I saw this thing today', and we tell the story, and we maybe don't consciously realise but we're saying it to get it off our chest" (#14, 4th year student).

For peer review only - http://bmjopen.bmj.com/site/about/guidelines.xhtml

Beneficial work cultures, including camaraderie, humour and team dynamics, cultivated a sense of solidarity whilst working in challenging circumstances. Rewarding aspects of working in the medical profession were also highlighted. In particular, participants suggested that practising medicine had provided them with a sense of purpose and identified the psychological benefits associated with treating patients.

> *"You are doing a job that is inherently helpful and rewarding and you feel good because of it and when something goes well, that buzz stays with you for a long time" (#14, 4th year student).*

Simultaneously, participants discussed how their usual methods for managing stress had been hindered by the COVID-19 pandemic. Many participants described feeling isolated and went extended periods without seeing their close friends and family, whilst several FY1s found it difficult to integrate into new work settings.

> *"When lockdown was happening, I couldn't leave, and I think it got to the point where I was getting quite stressed about if my parents get sick, or my grandparents got sick, I wouldn't be there" (#15, FY3).*

Awareness of formal support mechanisms

Participants felt that occupational health services had made extra efforts to support their wellbeing during COVID-19. Extra email communications, signposting to mental health services, wellbeing posters around hospitals and psychoeducational talks were some identified methods.

For peer review only - http://bmjopen.bmj.com/site/about/guidelines.xhtml

"During the pandemic there's been a real focus on mental health for all members of staff, be that porters, nurses, doctors, auxiliary staff or healthcare assistants, and there's been a real effort in reaching out to people" (#4, FY1).

However, some participants felt that they would have benefitted from receiving further support, such as more opportunities for rest and recovery, peer support and more accessible mental health services.

"I think X university do it, they have mental health days where you can sort of, no questions asked, 'I'm not going in today', and you can just email them and say that, and you have a certain number throughout the year" (#8, 5th year student).

Of the participants that had previously met with occupational services for their mental health, a majority suggested that they had not found it helpful. Participants felt that occupational services were more concerned about what this meant for their academic progression or work availability, rather than on offering them support. Some participants felt their negative experiences in dealing with occupational health services had discouraged them from accessing help in the future.

"It was occupational health, and I had a meeting with her, and she was very, very condescending (...), I think that was quite a rare experience, but it's definitely tainted my view of disclosing mental health issues" (#6, FY1).

Around half of the sample indicated that they would be concerned about career implications if they disclosed a mental health problem. Some participants felt this may affect their future

For peer review only - http://bmjopen.bmj.com/site/about/guidelines.xhtml

employability. Moreover, a couple of participants indicated reluctance to disclose a mental health problem to their general practitioner, stating concerns about the confidentiality of their medical record.

> *"It's something that you shouldn't be worried about because it shouldn't be too different to any other health condition, but it would worry me, I think it does worry me a little bit, so when I got the job with the NHS, I didn't put that down" (#14, 4th year student).*

Whilst most participants felt supported by their clinical colleagues, tutors and peers, attitudes towards administrative staff were more variable. Participants reported feeling underappreciated and inadequately supported by management, suggesting that they lacked insight into the pressures involved in practicing medicine.

> *"The senior doctors will try to help you out and give you advice, but not from the actual hospital management, rota co-ordinators, they have no clue, everything's like a number for them, it's almost like the business side of things so they don't know the pressures of actually working for the hospital" (#11, FY1).*

For peer review only

Discussion

Medical students and junior doctors discussed high levels of occupational stress which impacted on their wellbeing during the COVID-19 pandemic. Various stressors inherent to practising medicine, such as significant workloads and emotional challenges, were identified as well as organisational difficulties associated with the pandemic. Issues pertaining to resourcing, staffing, preparedness and adapting to new training formats exacerbated participants' work-stress during the pandemic. Extra efforts to support medical students and junior doctors during the pandemic were noted, however, participants also perceived barriers to accessing mental health support. These included a lack of clarity on their career implications, concerns about adding to colleagues' workloads, stigma in the profession and previous negative experiences with occupational health services.

Medical cultures which promote presenteeism and personality traits including perfectionism, self-criticism, and competitiveness were viewed as influencing participants' work stress. Previously, a 'hidden curriculum' within medical training has been described, where trainees are indoctrinated with social norms that may be maladaptive [22 24]. These norms place medical trainees under pressure to accept insufficient working conditions [24 25 49 50], or feel guilty for letting patients down, and burdening their colleagues with extra work if they themselves need time off [6 51 52]. At the same time, doctors receive validation from their peers for going above and beyond for their patients, producing an environment which sustains burnout [24].

Participants discussed factors that might prevent them from accessing help for their mental wellbeing, which echoed previous findings where medical cultures of secrecy and distrust were identified, as well as concerns around being perceived as weak [53 54]. Furthermore, participants worried about the career implications of disclosing a mental health problem to

For peer review only - http://bmjopen.bmj.com/site/about/guidelines.xhtml

occupational and health services, indicating the need for improved clarity and transparency in this process. A previous study on depression, stigma and suicidal ideation in medical students revealed that 53% of respondents who reported depressive symptoms worried that revealing their illness would be risky for their career [55]. Participants also described negative experiences interacting with occupational services, which deterred them from accessing mental health support in the future.

All of the research participants recognised progress towards promoting and managing mental health within the medical profession. Participants described how attitudes towards mental health had improved acutely over the last 18 months, which may reflect extra efforts to support medical workers during the COVID-19 pandemic [56]. In line with previous findings [57] [58], the medical students and junior doctors noted the importance of peer support. However, a majority of participants indicated that the pandemic had limited their access to support and coping strategies which would usually benefit their wellbeing. Despite this, participants suggested that working in the medical profession had been somewhat protective for their mental wellbeing during COVID-19, in that it is an inherently helpful, rewarding and sociable profession.

To our knowledge, no qualitative studies have simultaneously investigated the mental health of medical students and junior doctors during the COVID-19 pandemic. A further study strength is that an independent researcher carried out the interviews and analysis, adding an extra layer of privacy protection and strong reassurance of participant confidentiality. This study accessed a very unique cohort of medical students and junior doctors working in unprecedented times for the medical profession, therefore adding to the current sparse literature [59]. Despite this, the representativeness and transferability of the findings may be

For peer review only - http://bmjopen.bmj.com/site/about/guidelines.xhtml
For peer review only

limited, as most participants were males and of a white-British ethnicity. This is of particular concern as women, and ethnically diverse medics were reported to have suffered from higher rates of anxiety, depression and stress during the COVID-19 pandemic [60-62]. Additionally, whilst snowball sampling proved a useful recruitment technique, selection and response biases cannot be excluded [63], and the sample may not represent the experience of medics across the UK.

Protective measures should be implemented to minimise medical students' and junior doctors' occupational stress. These might include ensuring that workers only work their scheduled hours, encouraging more autonomy in shift allocation, and consistently monitoring their wellbeing [64]. In particular, communications should be aimed towards leadership structures, to promote supportive and compassionate cultures, and to role model setting boundaries around work-life balance [6 22]. These practices should be in place from medical school onwards, when first year students begin to be socialised into the profession [24]. Finally, individuals who need to take time off should feel able to do so and be made aware of the support systems in place [56].

The importance of peer support was clear in the findings. Consequently, interventions which foster peer support must be considered, and existing support from colleagues, supervisors and teams should be utilised [22]. Elsewhere, good practices have included 'buddying-up' schemes to reduce isolation and peer support groups where medics can discuss the emotional impact of their work [56 65]. Furthermore, the career implications of disclosing mental health problems must be clarified. Educational and training interventions may be adopted, which can encourage help seeking behaviours and boost medics' resilience [64 66 67].

For peer review only - http://bmjopen.bmj.com/site/about/guidelines.xhtml

Given the exploratory nature of this research, larger qualitative and quantitative studies are required to determine the generalisability and transferability of the findings. In particular, studies investigating perceived differences in mental health problems by sociodemographic, educational and geographical factors are warranted. Future studies may benefit from exploring the views of educational, occupational and healthcare management staff, to add other perspectives on the factors affecting this cohort's wellbeing. Further validation of the findings could also be added using observational research in order to provide more impetus for quality improvement interventions to be implemented.

As we move away from the pressures of COVID-19 pandemic, the systemic and cultural issues within medicine remain highly relevant. This paper advocates for improvement to working conditions, more communication and connection within teams, and for cultural change, in medical education and in health services.

Acknowledgements: The authors thank all the participants for sharing their experiences.

Author contributions: JT - conceived and designed the study; conducted the participant interviews, data analyses and drafted the manuscript; ED –contributed to the interpretation of the results and data analysis and with critical revisions of the manuscript.

Data statement: The data is not currently available for further use.

Ethical approval: Ethical approval was obtained from the University of Glasgow, School of Social & Political Sciences committee (PGT/SPS/2021/035/GLOB). Patients or the public were not involved in the design and conduct of this study.

Funding statement: ED acknowledges funding from the Medical Research Council (MC_UU_00022/2) and the Chief Scientist Office (SPHSU17).

For peer review only - http://bmjopen.bmj.com/site/about/guidelines.xhtml

Competing interests: An initial sample of participants were recruited through personal contacts of the lead researcher and the remainder were recruited using snowball sampling. ED declares no competing interests.

For peer review only

For peer review only - http://bmjopen.bmj.com/site/about/guidelines.xhtml

References

1. Lai CC, Shih TP, Ko WC, et al. Severe acute respiratory syndrome coronavirus 2 (SARS-CoV-2) and coronavirus disease-2019 (COVID-19): The epidemic and the challenges. *Int J Antimicrob Agents* 2020;55(3):105924. doi: 10.1016/j.ijantimicag.2020.105924 [published Online First: 2020/02/23]
2. Thome J, Deloyer J, Coogan AN, et al. The impact of the early phase of the COVID-19 pandemic on mental-health services in Europe. *World J Biol Psychiatry* 2020:1-10. doi: 10.1080/15622975.2020.1844290 [published Online First: 2020/11/05]
3. Menon A, Klein EJ, Kollars K, et al. Medical Students Are Not Essential Workers: Examining Institutional Responsibility During the COVID-19 Pandemic. *Acad Med* 2020;95(8):1149-51. doi: 10.1097/ACM.0000000000003478 [published Online First: 2020/04/30]
4. Arumi A, Bulbena-Cabre A, Bulbena A. First Person Account COVID 19 Delirium in a Doctor: When Death Stalks the Mind. *Front Psychiatry* 2021;12:626648. doi: 10.3389/fpsyt.2021.626648 [published Online First: 2021/02/16]
5. Michaeli D, Keough G, Perez-Dominguez F, et al. Medical education and mental health during COVID-19: a survey across 9 countries. *Int J Med Educ* 2022;13:35-46. doi: 10.5116/ijme.6209.10d6 [published Online First: 2022/03/01]
6. Spiers J, Buszewicz M, Chew-Graham C, et al. What challenges did junior doctors face while working during the COVID-19 pandemic? A qualitative study. *BMJ Open* 2021;11(12):e056122. doi: 10.1136/bmjopen-2021-056122 [published Online First: 2021/12/15]
7. Rainbow S, Dorji T. Impact of COVID-19 on medical students in the United Kingdom. *GERMS* 2020;10(3):240-43. doi: 10.18683/germs.2020.1210

For peer review only - http://bmjopen.bmj.com/site/about/guidelines.xhtml

8. Papapanou M, Routsi E, Tsamakis K, et al. Medical education challenges and innovations during COVID-19 pandemic. *Postgrad Med J* 2022;98(1159):321-27. doi: 10.1136/postgradmedj-2021-140032 [published Online First: 2021/03/31]

9. Ali A, Staunton M, Quinn A, et al. Exploring medical students' perceptions of the challenges and benefits of volunteering in the intensive care unit during the COVID-19 pandemic: a qualitative study. *BMJ Open* 2021;11(12):e055001. doi: 10.1136/bmjopen-2021-055001 [published Online First: 2021/12/26]

10. Harvey A. Covid-19: medical schools given powers to graduate final year students early to help NHS. *BMJ* 2020;368:m1227. doi: 10.1136/bmj.m1227 [published Online First: 2020/03/29]

11. Monrouxe LV, Bullock A, Gormley G, et al. New graduate doctors' preparedness for practice: a multistakeholder, multicentre narrative study. *BMJ Open* 2018;8(8):e023146. doi: 10.1136/bmjopen-2018-023146 [published Online First: 2018/08/31]

12. Pandey U, Corbett G, Mohan S, et al. Anxiety, Depression and Behavioural Changes in Junior Doctors and Medical Students Associated with the Coronavirus Pandemic: A Cross-Sectional Survey. *J Obstet Gynaecol India* 2020:1-5. doi: 10.1007/s13224-020-01366-w [published Online First: 2020/09/30]

13. Dunning A, Teoh K, Martin J, et al. Relationship between working conditions and psychological distress experienced by junior doctors in the UK during the COVID-19 pandemic: a cross-sectional survey study. *BMJ Open* 2022;12(8):e061331. doi: 10.1136/bmjopen-2022-061331 [published Online First: 2022/08/24]

14. Pappa S, Ntella V, Giannakas T, et al. Prevalence of depression, anxiety, and insomnia among healthcare workers during the COVID-19 pandemic: A systematic review and

For peer review only

For peer review only - http://bmjopen.bmj.com/site/about/guidelines.xhtml

meta-analysis. *Brain Behav Immun* 2020;88:901-07. doi: 10.1016/j.bbi.2020.05.026 [published Online First: 2020/05/22]

15. Farrell SM, Kadhum M, Lewis T, et al. Wellbeing and burnout amongst medical students in England. *Int Rev Psychiatry* 2019;31(7-8):579-83. doi: 10.1080/09540261.2019.1675960 [published Online First: 2019/11/07]

16. Moir F, Yielder J, Sanson J, et al. Depression in medical students: current insights. *Adv Med Educ Pract* 2018;9:323-33. doi: 10.2147/AMEP.S137384 [published Online First: 2018/05/17]

17. Henning MA, Sollers J, Strom JM, et al. Junior doctors in their first year: mental health, quality of life, burnout and heart rate variability. *Perspect Med Educ* 2014;3(2):136-43. doi: 10.1007/s40037-013-0075-y [published Online First: 2014/04/08]

18. Bhugra D, Sauerteig SO, Bland D, et al. A descriptive study of mental health and wellbeing of doctors and medical students in the UK. *Int Rev Psychiatry* 2019;31(7-8):563-68. doi: 10.1080/09540261.2019.1648621 [published Online First: 2019/08/29]

19. Royal College of Physicians. Being a junior doctor: Experiences from the front line of the NHS. *London* 2016

20. Zeng W, Chen R, Wang X, et al. Prevalence of mental health problems among medical students in China: A meta-analysis. *Medicine (Baltimore)* 2019;98(18):e15337. doi: 10.1097/MD.0000000000015337 [published Online First: 2019/05/03]

21. Zivanovic R, McMillan J, Lovato C, et al. Death by Suicide among Canadian Medical Students: A National Survey-Based Study. *Can J Psychiatry* 2018;63(3):178-81. doi: 10.1177/0706743717746663 [published Online First: 2018/03/08]

22. Riley R, Buszewicz M, Kokab F, et al. Sources of work-related psychological distress experienced by UK-wide foundation and junior doctors: a qualitative study. *BMJ*

For peer review only

Open 2021;11(6):e043521. doi: 10.1136/bmjopen-2020-043521 [published Online First: 2021/06/25]

23. Riley R, Spiers J, Buszewicz M, et al. What are the sources of stress and distress for general practitioners working in England? A qualitative study. *BMJ Open* 2018;8(1):e017361. doi: 10.1136/bmjopen-2017-017361 [published Online First: 2018/01/13]

24. Wong AMF. Beyond burnout: looking deeply into physician distress. *Can J Ophthalmol* 2020;55(3 Suppl 1):7-16. doi: 10.1016/j.jcjo.2020.01.014 [published Online First: 2020/03/25]

25. Lock FK, Carrieri D. Factors affecting the UK junior doctor workforce retention crisis: an integrative review. *BMJ Open* 2022;12(3):e059397. doi: 10.1136/bmjopen-2021-059397 [published Online First: 2022/03/31]

26. Zis P, Artemiadis A, Bargiotas P, et al. Medical Studies during the COVID-19 Pandemic: The Impact of Digital Learning on Medical Students' Burnout and Mental Health. *Int J Environ Res Public Health* 2021;18(1) doi: 10.3390/ijerph18010349 [published Online First: 2021/01/21]

27. Essadek A, Gressier F, Robin M, et al. Mental health of medical students during the COVID19: Impact of studies years. *J Affect Disord Rep* 2022;8:100318. doi: 10.1016/j.jadr.2022.100318 [published Online First: 2022/02/16]

28. O'Byrne L, Gavin B, Adamis D, et al. Levels of stress in medical students due to COVID-19. *J Med Ethics* 2021 doi: 10.1136/medethics-2020-107155 [published Online First: 2021/03/05]

29. Warren J, Plunkett E, Rudge J, et al. Trainee doctors' experiences of learning and well-being while working in intensive care during the COVID-19 pandemic: a qualitative

For peer review only - http://bmjopen.bmj.com/site/about/guidelines.xhtml

study using appreciative inquiry. *BMJ Open* 2021;11(5):e049437. doi: 10.1136/bmjopen-2021-049437 [published Online First: 2021/05/27]

30. Byrne JP, Creese J, Matthews A, et al. '...the way it was staffed during COVID is the way it should be staffed in real life...': a qualitative study of the impact of COVID-19 on the working conditions of junior hospital doctors. *BMJ Open* 2021;11(8):e050358. doi: 10.1136/bmjopen-2021-050358 [published Online First: 2021/08/11]
31. Olah B, Radi BM, Kosa K. Barriers to Seeking Mental Help and Interventions to Remove Them in Medical School during the COVID-19 Pandemic: Perspectives of Students. *Int J Environ Res Public Health* 2022;19(13) doi: 10.3390/ijerph19137662 [published Online First: 2022/07/10]
32. Kromydas T, Green M, Craig P, et al. Comparing population-level mental health of UK workers before and during the COVID-19 pandemic: a longitudinal study using Understanding Society. *Journal of Epidemiology and Community Health* 2022;76(6):527. doi: 10.1136/jech-2021-218561
33. Mutambudzi M, Niedwiedz C, Macdonald EB, et al. Occupation and risk of severe COVID-19: prospective cohort study of 120 075 UK Biobank participants. *Occupational and Environmental Medicine* 2020
34. Griffin L, Riley R. Exploring the psychological impact of working during COVID-19 on medical and nursing students: a qualitative study. *BMJ Open* 2022;12(6):e055804. doi: 10.1136/bmjopen-2021-055804 [published Online First: 2022/06/24]
35. Wilkinson C, Finn G, Crampton P. Responsibility with a Safety Net: Exploring the Medical Student to Junior Doctor Transition During COVID-19. *Medical Science Educator* 2021;32(1):121-29. doi: 10.1007/s40670-021-01476-8
36. Petrino R, Riesgo LG, Yilmaz B. Burnout in emergency medicine professionals after 2 years of the COVID-19 pandemic: a threat to the healthcare system? *Eur J Emerg*

For peer review only - http://bmjopen.bmj.com/site/about/guidelines.xhtml

Med 2022;29(4):279-84. doi: 10.1097/MEJ.0000000000000952 [published Online First: 2022/05/28]

37. Zhou T, Xu C, Wang C, et al. Burnout and well-being of healthcare workers in the post-pandemic period of COVID-19: a perspective from the job demands-resources model. *BMC Health Serv Res* 2022;22(1):284. doi: 10.1186/s12913-022-07608-z [published Online First: 2022/03/04]

38. Palinkas LA, Horwitz SM, Green CA, et al. Purposeful Sampling for Qualitative Data Collection and Analysis in Mixed Method Implementation Research. *Adm Policy Ment Health* 2015;42(5):533-44. doi: 10.1007/s10488-013-0528-y [published Online First: 2013/11/07]

39. Noy C. Sampling Knowledge: The Hermeneutics of Snowball Sampling in Qualitative Research. *International Journal of Social Research Methodology* 2008;11(4):327-44. doi: 10.1080/13645570701401305

40. Naderifar M, Goli H, Ghaljaie F. Snowball Sampling: A Purposeful Method of Sampling in Qualitative Research. *Strides in Development of Medical Education* 2017;14(3) doi: 10.5812/sdme.67670

41. Sanjari M, Bahramnezhad F, Fomani FK, et al. Ethical challenges of researchers in qualitative studies: the necessity to develop a specific guideline. *J Med Ethics Hist Med* 2014;7:14. [published Online First: 2014/12/17]

42. Antoni A, Beer H. Research Impact as Care: Re-Conceptualizing Research Impact From an Ethics of Care Perspective. *Business Ethics and Care in Organizations* 2019

43. Schofield G, Dittborn M, Selman LE, et al. Defining ethical challenge(s) in healthcare research: a rapid review. *BMC Med Ethics* 2021;22(1):135. doi: 10.1186/s12910-021-00700-9 [published Online First: 2021/10/01]

For peer review only

44. Williamson AE, Burns N. The safety of researchers and participants in primary care qualitative research. *Br J Gen Pract* 2014;64(621):198-200. doi: 10.3399/bjgp14X679480 [published Online First: 2014/04/02]

45. Keen S, Lomeli-Rodriguez M, Joffe H. From Challenge to Opportunity: Virtual Qualitative Research During COVID-19 and Beyond. *Int J Qual Methods* 2022;21:16094069221105075. doi: 10.1177/16094069221105075 [published Online First: 2022/06/14]

46. O'Brien BC, Harris IB, Beckman TJ, et al. Standards for Reporting Qualitative Research: A Synthesis of Recommendations. *Academic Medicine* 2014;89(9)

47. Braun V, Clarke V. Reflecting on reflexive thematic analysis. *Qualitative Research in Sport, Exercise and Health* 2019;11(4)

48. Palinkas LA. Qualitative and mixed methods in mental health services and implementation research. *J Clin Child Adolesc Psychol* 2014;43(6):851-61. doi: 10.1080/15374416.2014.910791 [published Online First: 2014/10/29]

49. Wainwright E, Fox F, Breffni T, et al. Coming back from the edge: a qualitative study of a professional support unit for junior doctors. *BMC Med Educ* 2017;17(1):142. doi: 10.1186/s12909-017-0978-0 [published Online First: 2017/08/25]

50. Smith SE, Tallentire VR, Pope LM, et al. Foundation Year 2 doctors' reasons for leaving UK medicine: an in-depth analysis of decision-making using semistructured interviews. *BMJ Open* 2018;8(3):e019456. doi: 10.1136/bmjopen-2017-019456 [published Online First: 2018/03/04]

51. Creese J, Byrne JP, Conway E, et al. "We All Really Need to just Take a Breath": Composite Narratives of Hospital Doctors' Well-Being during the COVID-19 Pandemic. *Int J Environ Res Public Health* 2021;18(4) doi: 10.3390/ijerph18042051 [published Online First: 2021/03/07]

For peer review only

52. Henderson M, Brooks SK, del Busso L, et al. Shame! Self-stigmatisation as an obstacle to sick doctors returning to work: a qualitative study: Table 1. *BMJ Open* 2012;2(5) doi: 10.1136/bmjopen-2012-001776

53. Bianchi EF, Bhattacharyya MR, Meakin R. Exploring senior doctors' beliefs and attitudes regarding mental illness within the medical profession: a qualitative study. *BMJ Open* 2016;6(9):e012598. doi: 10.1136/bmjopen-2016-012598 [published Online First: 2016/09/18]

54. Waugh W, Lethem C, Sherring S, et al. Exploring experiences of and attitudes towards mental illness and disclosure amongst health care professionals: a qualitative study. *J Ment Health* 2017;26(5):457-63. doi: 10.1080/09638237.2017.1322184 [published Online First: 2017/05/11]

55. Schwenk TL, Davis L, Wimsatt LA. Depression, stigma, and suicidal ideation in medical students. *JAMA* 2010;304(11):1181-90. doi: 10.1001/jama.2010.1300 [published Online First: 2010/09/16]

56. BMA. The mental health and wellbeing of the medical workforce – now and beyond COVID-19. 2021

57. Fisher EB, Ayala GX, Ibarra L, et al. Contributions of Peer Support to Health, Health Care, and Prevention: Papers from Peers for Progress. *Ann Fam Med* 2015;13 Suppl 1:S2-8. doi: 10.1370/afm.1852 [published Online First: 2015/08/26]

58. Moir F, Henning M, Hassed C, et al. A Peer-Support and Mindfulness Program to Improve the Mental Health of Medical Students. *Teach Learn Med* 2016;28(3):293-302. doi: 10.1080/10401334.2016.1153475 [published Online First: 2016/04/20]

59. Kerins J, Hamilton AL, Pringle J, et al. Exploring the impact of the COVID-19 pandemic on doctors' core workplace needs: a qualitative study of internal medicine trainees in

For peer review only - http://bmjopen.bmj.com/site/about/guidelines.xhtml

Scotland. *BMJ Open* 2021;11(6):e053506. doi: 10.1136/bmjopen-2021-053506 [published Online First: 2021/07/02]

60. Morgan R, Tan HL, Oveisi N, et al. Women healthcare workers' experiences during COVID-19 and other crises: A scoping review. *Int J Nurs Stud Adv* 2022;4:100066. doi: 10.1016/j.ijnsa.2022.100066 [published Online First: 2022/02/08]

61. Regenold N, Vindrola-Padros C. Gender Matters: A Gender Analysis of Healthcare Workers' Experiences during the First COVID-19 Pandemic Peak in England. *Social Sciences* 2021;10(2) doi: 10.3390/socsci10020043

62. Qureshi I, Gogoi M, Wobi F, et al. Healthcare Workers From Diverse Ethnicities and Their Perceptions of Risk and Experiences of Risk Management During the COVID-19 Pandemic: Qualitative Insights From the United Kingdom-REACH Study. *Frontiers in Medicine* 2022;9 doi: 10.3389/fmed.2022.930904

63. Pannucci CJ, Wilkins EG. Identifying and avoiding bias in research. *Plast Reconstr Surg* 2010;126(2):619-25. doi: 10.1097/PRS.0b013e3181de24bc [published Online First: 2010/08/04]

64. Vizheh M, Qorbani M, Arzaghi SM, et al. The mental health of healthcare workers in the COVID-19 pandemic: A systematic review. *J Diabetes Metab Disord* 2020:1-12. doi: 10.1007/s40200-020-00643-9 [published Online First: 2020/11/03]

65. Behrman S, Baruch N, Stegen G. Peer support for junior doctors: a positive outcome of the COVID-19 pandemic? *Future Healthc J* 2020;7(3):e64-e66. doi: 10.7861/fhj.2020-0069 [published Online First: 2020/10/24]

66. Yiu V. Supporting the well-being of medical students. *CMAJ* 2005;172(7):889-90. doi: 10.1503/cmaj.050126 [published Online First: 2005/03/30]

67. Steen M, Othman SME, Briley A, et al. Self-compassion Education for Health Professionals (Nurses and Midwives): Protocol for a Sequential Explanatory Mixed

For peer review only - http://bmjopen.bmj.com/site/about/guidelines.xhtml

Methods Study. *JMIR Res Protoc* 2022;11(1):e34372. doi: 10.2196/34372 [published Online First: 2021/12/02]

Figure Legends

Figure 1**.** Thematic Map

For peer review only

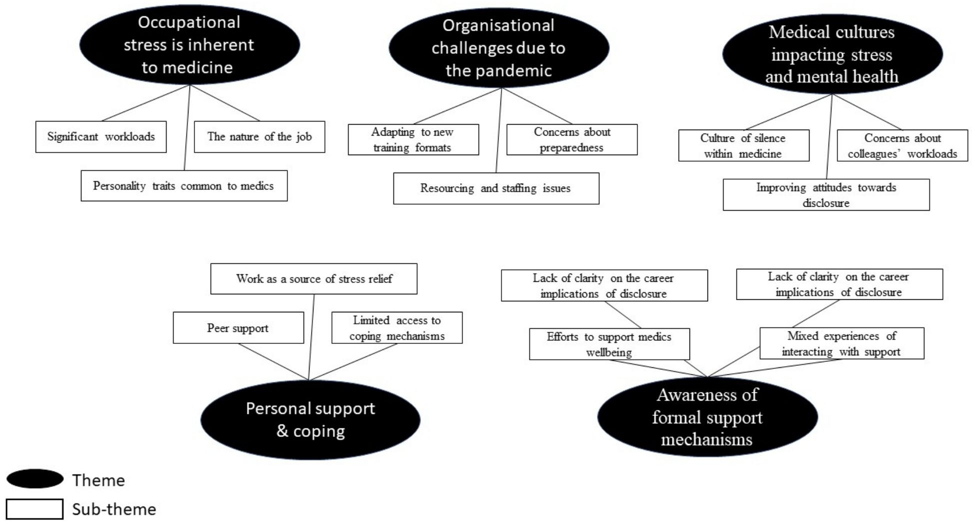

Figure 1. Thematic Map

338x190mm (96 x 96 DPI)

Interview Guide

A qualitative study to explore UK medical students' and junior doctors' experiences of occupational stress and mental health during the COVID-19 pandemic.

Warm up questions

How are you today?

How has work/university been recently?

Have you got any plans this summer?

Main Questions

Experiences of occupational stress

Can you describe a time when you have experienced work-related stress?

Do you make any conscious efforts to reduce work-related stress?

Have you ever felt as though work-related stress has had an impact on your mental wellbeing?

Do you feel as though you are under more pressure than other occupations/degree subjects?

How has the pandemic influenced your experiences of stress/ work stress?

Has the pandemic limited your ability to manage your stress?

Is there anything that you think has been helping your mental health and wellbeing during the pandemic?

Is there anything you have done to try to relieve stress during the pandemic?

Attitudes towards mental illness

Do you feel as though working in the health profession increases or reduces your risk of suffering from a mental illness? – Discuss risk factors and protective factors

Would you agree with the perception that only certain people suffer from mental illnesses? If so, what type of person? Any particular personality traits/ behaviours

How do you feel colleagues would view a co-worker taking leave for mental ill health compared to a physical condition? Why may this be the case?

For peer review only

Barriers to disclosure

Hypothetically if you were suffering from mentally ill health, would there be any factors that may prevent you from disclosing it?

Would you be concerned at all about job stability if you were disclosing a mental illness to a manager or colleagues?

Would you be concerned about experiencing stigma from co-workers if you disclosed a mental illness?

If you were suffering from a mental illness what support networks would be available for you? What would be your preferred route?

Would access to help services be limited by the COVID pandemic?

Cool down questions

Did you find everything okay with the interview?

Do you have any questions or anything you'd like to clarify?

Are you doing anything after this?

Thank participants for their time and for meeting with me.

For peer review only

Standards for Reporting Qualitative Research (SRQR)*

http://www.equator-network.org/reporting-guidelines/srqr/

Title and abstract

	Page/line no(s).
**Title** - Concise description of the nature and topic of the study Identifying the study as qualitative or indicating the approach (e.g., ethnography, grounded theory) or data collection methods (e.g., interview, focus group) is recommended	Page 2, line 3
**Abstract** - Summary of key elements of the study using the abstract format of the intended publication; typically includes background, purpose, methods, results, and conclusions	Page 3, line 3

Introduction

**Problem formulation** - Description and significance of the problem/phenomenon studied; review of relevant theory and empirical work; problem statement	Page 5, Line 5
**Purpose or research question** - Purpose of the study and specific objectives or questions	Page 7 Line 17

Methods

**Qualitative approach and research paradigm** - Qualitative approach (e.g., ethnography, grounded theory, case study, phenomenology, narrative research) and guiding theory if appropriate; identifying the research paradigm (e.g., postpositivist, constructivist/ interpretivist) is also recommended; rationale**	Page 8, line 54
**Researcher characteristics and reflexivity** - Researchers' characteristics that may influence the research, including personal attributes, qualifications/experience, relationship with participants, assumptions, and/or presuppositions; potential or actual interaction between researchers' characteristics and the research questions, approach, methods, results, and/or transferability	Page 7, line 51
**Context** - Setting/site and salient contextual factors; rationale**	Page 8, line 4
**Sampling strategy** - How and why research participants, documents, or events were selected; criteria for deciding when no further sampling was necessary (e.g., sampling saturation); rationale**	Page 7, line 49
**Ethical issues pertaining to human subjects** - Documentation of approval by an appropriate ethics review board and participant consent, or explanation for lack thereof; other confidentiality and data security issues	Page 8, line 6
**Data collection methods** - Types of data collected; details of data collection procedures including (as appropriate) start and stop dates of data collection and analysis, iterative process, triangulation of sources/methods, and modification of procedures in response to evolving study findings; rationale**	Page 8, line 24

For peer review only

For peer review only - http://bmjopen.bmj.com/site/about/guidelines.xhtml

**Data collection instruments and technologies** - Description of instruments (e.g., interview guides, questionnaires) and devices (e.g., audio recorders) used for data collection; if/how the instrument(s) changed over the course of the study	Page 8, line 29
**Units of study** - Number and relevant characteristics of participants, documents, or events included in the study; level of participation (could be reported in results)	Page 9, line 18
**Data processing** - Methods for processing data prior to and during analysis, including transcription, data entry, data management and security, verification of data integrity, data coding, and anonymization/de-identification of excerpts	Page 8, line 29
**Data analysis** - Process by which inferences, themes, etc., were identified and developed, including the researchers involved in data analysis; usually references a specific paradigm or approach; rationale**	Page 8, line 54
**Techniques to enhance trustworthiness** - Techniques to enhance trustworthiness and credibility of data analysis (e.g., member checking, audit trail, triangulation); rationale**	Page 9, line 3

Results/findings

**Synthesis and interpretation** - Main findings (e.g., interpretations, inferences, and themes); might include development of a theory or model, or integration with prior research or theory	Page 11, line 10
**Links to empirical data** - Evidence (e.g., quotes, field notes, text excerpts, photographs) to substantiate analytic findings	Page 11, Line 24

Discussion

**Integration with prior work, implications, transferability, and contribution(s) to the field -** Short summary of main findings; explanation of how findings and conclusions connect to, support, elaborate on, or challenge conclusions of earlier scholarship; discussion of scope of application/generalizability; identification of unique contribution(s) to scholarship in a discipline or field	Page 20, Line 4
**Limitations** - Trustworthiness and limitations of findings	Page 22, Line 1

Other

**Conflicts of interest** - Potential sources of influence or perceived influence on study conduct and conclusions; how these were managed	Page 24, Line 4
**Funding** - Sources of funding and other support; role of funders in data collection, interpretation, and reporting	Page 23, Line 56

*The authors created the SRQR by searching the literature to identify guidelines, reporting standards, and critical appraisal criteria for qualitative research; reviewing the reference lists of retrieved sources; and contacting experts to gain feedback. The SRQR aims to improve the transparency of all aspects of qualitative research by providing clear standards for reporting qualitative research.

For peer review only - http://bmjopen.bmj.com/site/about/guidelines.xhtml

**The rationale should briefly discuss the justification for choosing that theory, approach, method, or technique rather than other options available, the assumptions and limitations implicit in those choices, and how those choices influence study conclusions and transferability. As appropriate, the rationale for several items might be discussed together.

Reference:
O'Brien BC, Harris IB, Beckman TJ, Reed DA, Cook DA. **Standards for reporting qualitative research: a synthesis of recommendations.** *Academic Medicine*, Vol. 89, No. 9 / Sept 2014 DOI: 10.1097/ACM.0000000000000388

For peer review only

For peer review only - http://bmjopen.bmj.com/site/about/guidelines.xhtml
